# Room-temperature phosphorescence of a supercooled liquid: kinetic stabilisation by desymmetrisation†

**DOI:** 10.1039/d1sc03800a

**Published:** 2021-09-10

**Authors:** Mao Komura, Takuji Ogawa, Yosuke Tani

**Affiliations:** Department of Chemistry, Graduate School of Science, Osaka University 1-1 Machikaneyama Toyonaka Osaka 560-0043 Japan y-tani@chem.sci.osaka-u.ac.jp

## Abstract

Achieving organic room-temperature phosphorescence (RTP) in a solvent-free liquid state is a challenging task because the liquid state provides a less rigid environment than the crystal. Here, we report that an unsymmetrical heteroaromatic 1,2-diketone forms an organic RTP liquid. This diketone exists as a kinetically stable supercooled liquid, which resists crystallisation even under pricking or shearing stresses, and remains as a liquid for several months. The unsymmetrical diketone core is flexible, with eight distinct conformers possible, which prevents nucleation and growth for the liquid–solid transition. Interestingly, the thermodynamically stable crystalline solid-state was non-emissive. Thus, the RTP of the diketone was found to be liquiefaction-induced. Single-crystal X-ray structure analysis revealed that the diminished RTP of the crystal is due to insufficient intermolecular interactions and restricted access to an emissive conformer. Our work demonstrates that flexible unsymmetrical skeletons are promising motifs for bistable liquid–solid molecular systems, which are useful for the further development of stimuli-responsive materials that use phase transitions.

## Abstract 2

Metal-free, single-component, unsymmetrical 1,2-diketone exhibits liquefaction-induced room-temperature phosphorescence. Desymmetrisation provides the supercooled liquid with notable kinetic stability and phase-dependent phosphorescence properties.

## Graphical abstract

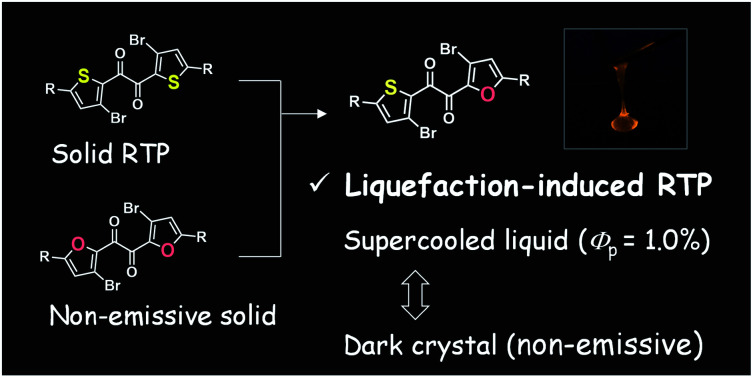

## Introduction

Room-temperature phosphorescence (RTP) is a unique luminescence phenomenon that is useful for a number of applications, including organic light-emitting diodes (OLEDs), bio-imaging, and encryption inks.^1^ In particular, metal-free organic RTP materials are desirable owing to their cost-effectiveness and low environmental burden.^2^ Most organic RTP luminophores require rigid crystal lattices to suppress non-radiative decay that quenches RTP.^3^ Since the optical properties of crystalline materials deteriorate through lattice defects, organic RTP materials that function in non-rigid and flexible molecular environments are desired.^4^

**Fig. 1:**
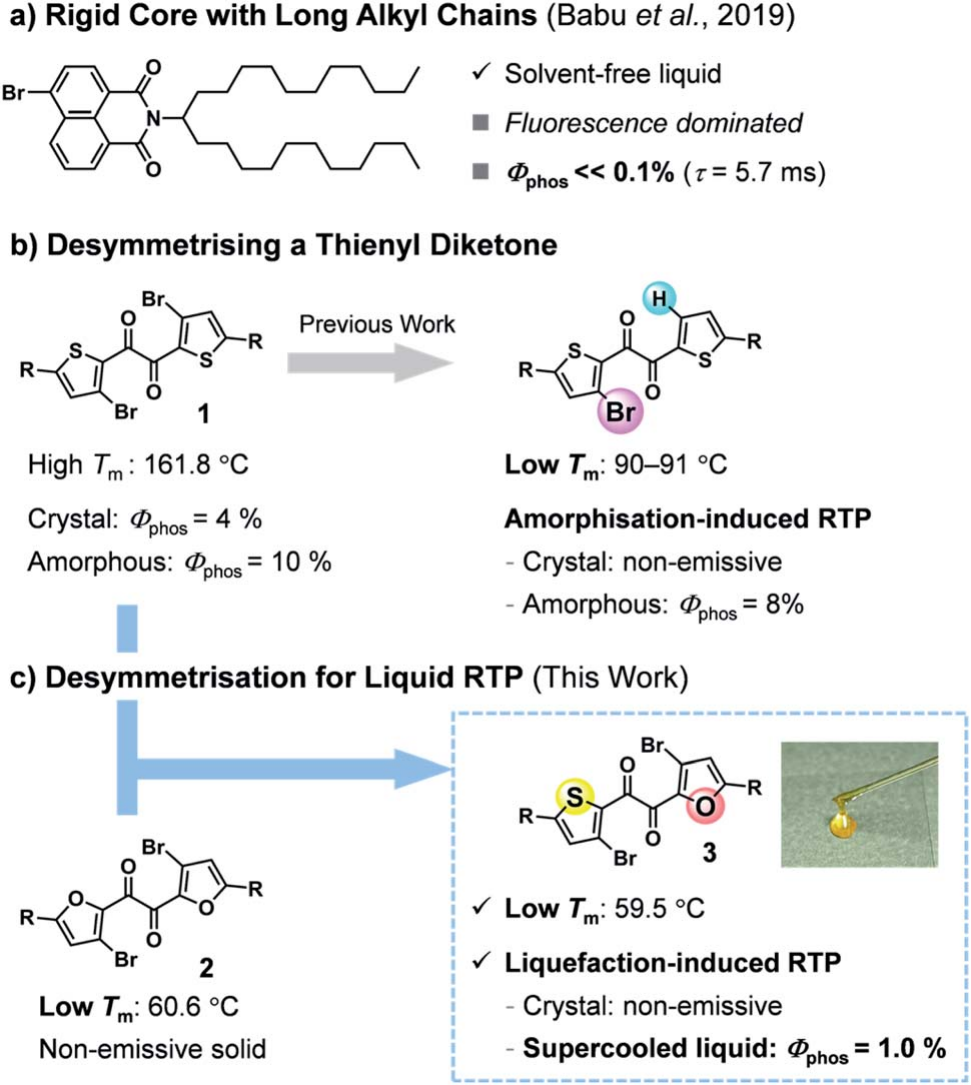
Strategy toward solvent-free liquid organic RTP. (a) Introducing long alkyl chains into a rigid bromonaphthalimide chromophore. (b and c) Desymmetrising *C*_2_-symmetrical thienyl diketones. The photographic image is of liquid **3** taken in room light. R = triisopropylsilyl, *T*_m_: melting point.

The liquid state is the most flexible of condensed materials. Solvent-free liquid chromophores are advantageous because they are easily processed and, unlike solution- or polymer-based materials, can exploit high chromophore densities.^5^ However, RTP is hardly observed in the liquid state, where molecules move more freely than in the solid state. To the best of our knowledge, only a pioneering two-component liquid system has been reported to show practical organic RTP.^6^ Babu *et al.* demonstrated that the introduction of a long, branched alkyl chain onto a rigid bromonaphthalimide chromophore produces a solvent-free liquid that exhibits visible RTP when mixed with carbonyl compounds; however, the molecular liquid itself showed fluorescence-dominated emission with a faint RTP (Fig. 1a). In addition, considering that a multicomponent (host-guest) system requires laborious optimisation (*e.g.*, choices of host and guest, their ratio, and fabrication procedure), single-component molecular RTP liquids are more convenient and advantageous.^7,8^

Previously, we found that thienyl diketone derivatives exhibit more efficient RTP in amorphous solid states than in the crystal, with phosphorescence quantum yields *Φ*_p_ of up to 10%, unaided by any metal heavy atom effect (Fig. 1b).^9^ Notably, unlike typical chromophores with rigid skeletons, the diketone skeleton is flexible, with five distinct conformers. Among them, a *trans*-planar (TP) conformer has two-fold intramolecular chalcogen bonds and is responsible for the efficient RTP in the amorphous solid states despite its minor proportion. We also demonstrated that desymmetrising *C*_2_ symmetrical diketone **1**—by replacing one bromine with hydrogen—reduced the intermolecular interactions in the crystals, dramatically lowered the melting point, and made the crystal non-emissive while maintaining the emissivity of the amorphous solid (Fig. 1b).^9*b*^ Consequently, the unsymmetrical diketone crystal showed, for the first time, turn-on RTP in response to mechanical stimuli in a metal-free organic molecule. These findings prompted us to expand the utility of the desymmetrisation approach.^10^

In this work, we envisaged that desymmetrisation provides a practical approach to achieving solvent-free liquid organic RTP (Fig. 1c). Unsymmetrical diketone **3** was designed to combine the characteristics of two *C*_2_-symmetrical diketones, namely the efficient RTP of **1** and the low melting point of furyl diketone **2**. Herein, we report that heteroaromatic 1,2-diketone **3** exhibits RTP in the solvent-free liquid state. Diketone **3** exists as a kinetically stable supercooled liquid (SCL), which is reluctant to crystallise even in the presence of seed crystals or under mechanical stimulation. To our surprise, the phosphorescence of **3** responds in an unusual way to a disorder-order phase transition; the SCL exhibited RTP in air, while the solid state was non-emissive despite its crystallinity. Moreover, the liquid exhibits a temperature-dependent phosphorescent colour shift (*i.e.*, thermochromism) that follows the thermal phase behaviour, including glass transition. These unique properties are attributed to the high conformational flexibility of **3**, demonstrating the usefulness of a phosphorescent molecular liquid with a flexible skeleton.

## Results and discussion

### Synthesis, characterisation, and photophysical properties of liquid **3**

Diketone **3** was synthesised by the cross-benzoin condensation of the corresponding aldehydes, followed by oxidation (see ESI† for details).^9^ After purification, overnight solvent removal at reduced pressure and 40 °C afforded **3** as a liquid (Fig. 1c); as-prepared **3** was confirmed to be pure and solvent-free by ^1^H NMR and ^13^C NMR spectroscopy, and elemental analysis. Liquid **3** is isotropic, as confirmed by X-ray diffractometry (XRD) and visual inspection under a polarising optical microscope (Fig. 4 and S1;†*vide infra*).

**Fig. 2:**
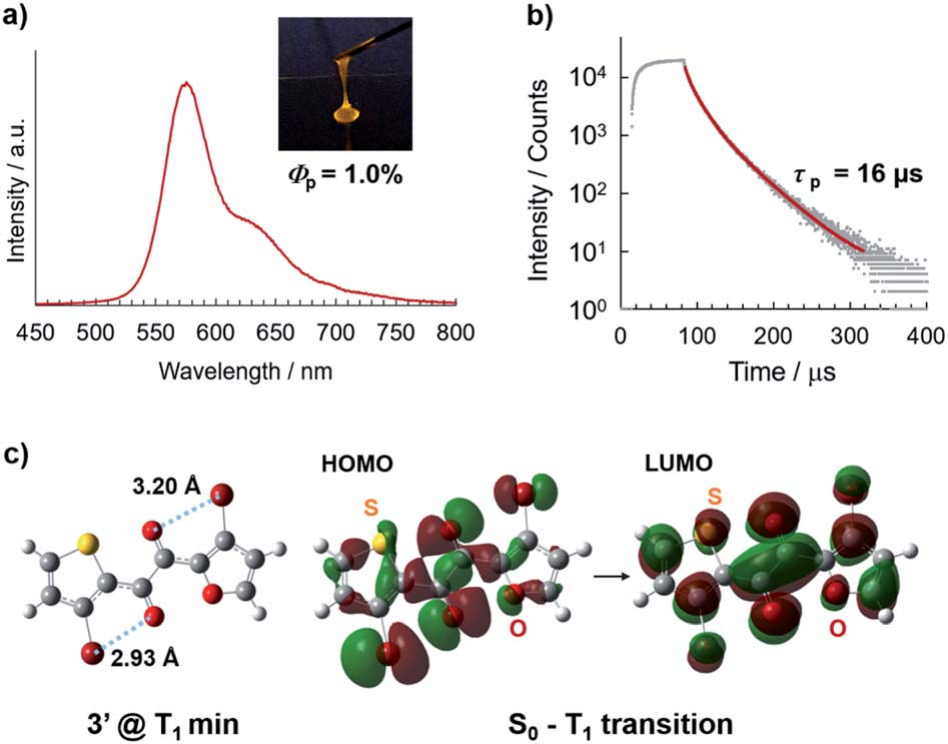
(a) Steady-state photoluminescence spectrum and (b) decay profile recorded at 570 nm of liquid **3** in air at room temperature (*λ*_ex_ = 368 nm). Inset: photographic image of liquid **3** when irradiated with UV light (365 nm). Absolute *Φ*_p_ was determined using an integrating sphere device. (c) Geometry of T_1_-minimum-energy TP conformer of **3′** and its Kohn–Sham HOMO and LUMO orbitals, calculated at the (TD-)UB3LYP-D3/6-311G(d) level of theory.

In air at room temperature, solvent-free liquid **3** exhibited orange phosphorescence without discernible fluorescence (Fig. 2a, b, and S5†); its steady-state photoluminescence (PL) spectrum exhibits an emission maximum at 574 nm with a PL lifetime of 16 μs, which does not include fast decay components of nanosecond order, consistent with phosphorescence-dominated emission without detectable amounts of fluorescence components. The *Φ*_p_ value was found to be 1.0%, which is the highest among single-component organic liquids.^6^ The RTP of liquid **3** likely originates from the TP conformer (Fig. 2c) because the PL spectrum of liquid **3** is quite similar to that of **1** in solution, whose RTP originates from the corresponding TP conformer (Fig. S7†).^9^ In addition, density functional theory (DFT) calculations at the UB3LYP-D3/6-311G(d) level of theory suggest that the most stable conformer of **3′** (in which the silyl groups in **3** are replaced by H) in the lowest triplet state (T_1_) is the TP conformer (Fig. 2c, S12, and Table S1†). Time-dependent (TD)-DFT calculations revealed that the T_1_–S_0_ radiative transition (phosphorescence) principally consists of the HOMO–LUMO transition (94%) with an electronic configuration with ^3^(n,π*) character, which is favourable for spin inversion according to El-Sayed's rule.^1^ Moreover, bromine atoms close to the carbonyl oxygen further promote phosphorescence by an intramolecularly directed heavy atom effect (Fig. 2c).^11^

### Photophysical properties of **3** in solution

The RTP of **3** was only visible in its solvent-free liquid state. In solution, **3** was virtually non-emissive, even under Ar (*Φ*_p_ = 0.04%, 2.2 × 10^−4^ M in cyclohexane).^9,12^ The PL spectrum of solution-phase **3** is in good agreement with that of the solvent-free liquid, which indicates that the emissive conformers are the same (*i.e.*, the TP conformer) for both states (Fig. S7†). However, the phosphorescence decay rate constants (*k*_p_), which reflect molecular electronic properties, are significantly different (Table 1).^13^ The liquid exhibits a *k*_p_ that is 50 times larger than the solution, which implies that the external heavy atom effect from adjacent molecules contributes in the condensed solvent-free liquid (Fig. S11†).^1^ In contrast, the non-radiative decay rate constants (*k*_nr_) of the solvent-free liquid in air and the degassed solution are not significantly different (Table 1). The RTP intensity of the liquid is only 1.47 times higher under N_2_ (Fig. S5†), suggesting that the liquid is sufficiently viscus to retard oxygen diffusion, thereby suppressing phosphorescence quenching. In support, a relatively high zero shear viscosity *η*_0_ = 45 ± 2 Pa s was observed for liquid **3** at 25 °C (Fig. S6†).^5,6,15^ Nonetheless, we emphasise that the RTP enhancement of liquid **3** principally originates from an increase in *k*_p_ rather than a decrease in *k*_nr_. These results demonstrate that RTP can be achieved by designing a system with a large *k*_p_, even in a flexible environment where molecular motion is not suppressed.

**Table 1:** Photophysical properties of **3** at room temperature

	*Φ*_p_	*τ*_p_/μs	*k*_p_ a/s^−1^	*k*_nr_ a/s^−1^
Solvent-free liquid^b^	1.0%^c^	16	650	62 000
Cyclohexane solution^d^	0.04%^e^	33	12	30 000

a Calculated according to the formulas: *k*_p_ = *Φ*_p_/*τ*_p_ and *k*_nr_ = (1 − *Φ*_p_)/*τ*_p_.

b In air.

c Absolute *Φ*_p_ determined using an integrating sphere device.

d 2.2 × 10^−4^ M under Ar.

e Relative *Φ*_p_ determined using quinine sulfate as the standard.^14^

### Structure and non-luminescence of **3** in the solid state

Diketone **3** remained liquid at room temperature for at least three months and eventually solidified. Surprisingly, the solid was non-emissive (Fig. S15†), although the crystalline nature of the solid was evident from the sharp diffraction peaks observed by powder XRD (Fig. 4, *vide infra*). Thus, **3** exhibits efficient RTP only in the kinetically stable supercooled liquid (SCL) and is invisible either in solution or in the crystalline state. The observed liquefaction-induced RTP highlights the uniqueness of **3**, considering that common organic RTP materials require rigid environments.^1–4^

**Fig. 3:**
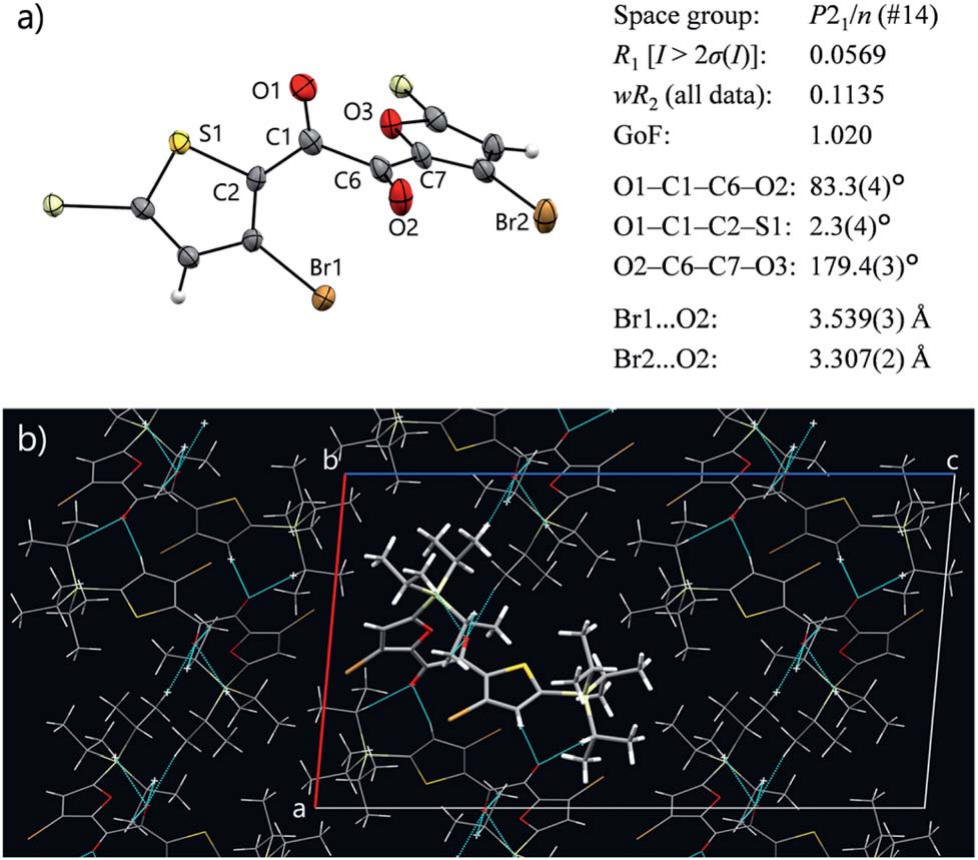
Single-crystal X-ray structure of **3**. (a) ORTEP drawing and selected crystallographic parameters. Isopropyl groups are omitted for clarity. Thermal ellipsoids are set at the 50% probability level. (b) Crystal packing seen along the *b* axis. The two Br atoms and the furan moiety have no short-contacts (dotted cyan lines).

To our delight, a single crystal of **3** suitable for X-ray structure analysis was obtained from a cooled MeOH solution. In the crystal, **3** has a skew conformation with a vicinal-dicarbonyl dihedral angle of 83.7(7)° (Fig. 3a). The powder XRD pattern simulated from the crystal structure is in good agreement with that of solid **3** obtained by solidification of SCL (Fig. S17†). Therefore, the molecular arrangements and conformations in the solid and the single crystal are comparable. Thus, the conformer in the solid state is different from the emitting conformer in the liquid state (TP conformer, Fig. 2c). The skew conformer has an electronic configuration that includes ^3^(π,π*) character, which is less favourable for phosphorescence (Fig. S14†). In addition, the bromine and oxygen atoms are clearly separated by long distances (Fig. 2c and 3a). Such differences in conformation provide reasons for why the same molecule emits RTP in the liquid but not in the crystal.

The crystal structure was further investigated from the perspective of intermolecular interactions to understand the origin of the non-luminescence of the solid state. Symmetrical diketone **1** exists in a skew conformation in the crystal;† however, it exhibits RTP owing to more-rigid crystal packing.^9*a*^ Previously, we created a non-emissive but isostructural crystal by desymmetrising **1** (Fig. 1b), and revealed that intermolecular short-contacts involving Br atoms are crucial for the crystal of **1** to exhibit RTP.^9*b*^ The Br atoms as well as the entire furan moiety have no short contacts in the crystal of **3**, which is disadvantageous for suppressing molecular motions (Fig. 3b). The rather isolated Br atoms also suggest that the external heavy atom effect, likely present in the liquid, is diminished owing to crystal packing. Moreover, Hirshfeld surface analysis of **3** and **1** was performed using CrystalExplorer software to quantitatively compare the intermolecular interactions in the crystals.^16^ An average normalised distance from nearest atoms is significantly longer for **3** than **1** (0.6468 *vs.* 0.5515 a.u., Fig. S16, see ESI† for further discussion), indicating weaker van der Waals interactions in crystalline **3**. Such insufficient intermolecular interactions, as well as the absence of the highly emissive TP conformer, are responsible for the lack of emissivity of solid **3**, despite its crystallinity.

### Stability of the SCL and SCL-to-solid phase transition behaviour

To gain insight into molecular designs that lead to liquefaction, we compared the solid–liquid phase-transition behaviour of the unsymmetrical diketone **3** with those of symmetrical diketones **1** and **2**. The melting points of **1**, **2**, and **3** are 161.8, 60.0, and 59.5 °C, respectively, which indicates that the thermodynamic liquid-phase stability of **3** is similar to that of **2**. Such a low melting point is beneficial for reactivating the RTP of **3** by heating and for applications in turn-on-type thermosensors.^17^

**Fig. 4:**
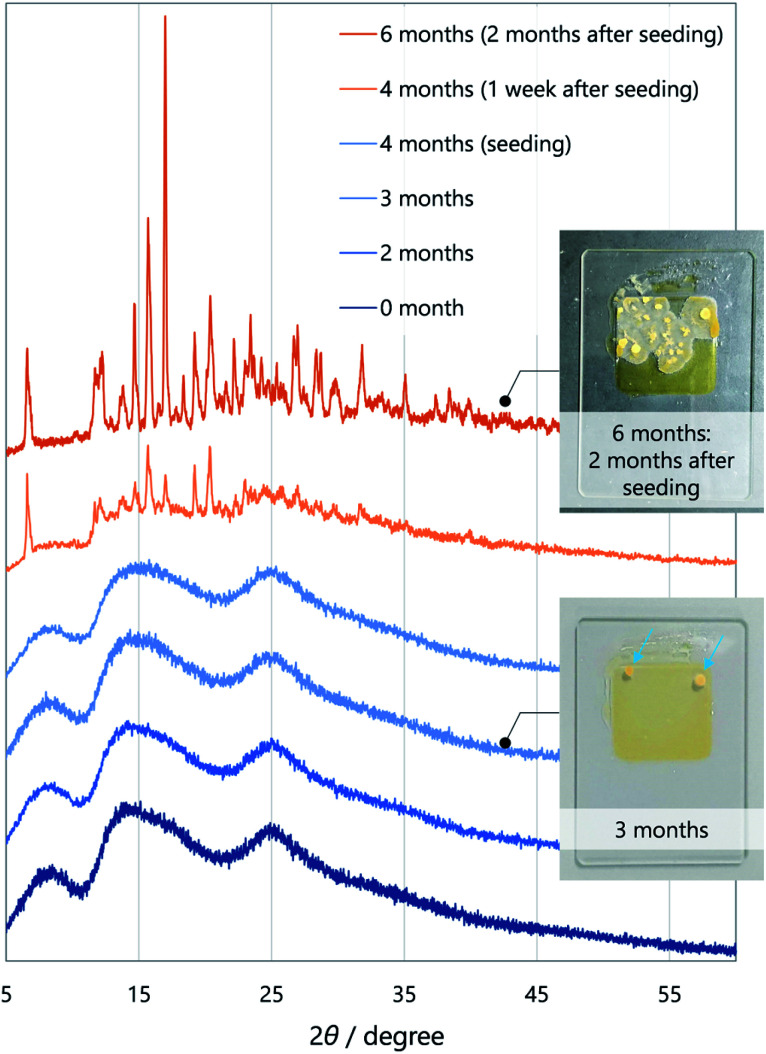
Crystallisation time-course of **3** followed by X-ray diffractometry. Inset: photographic images after three month (bottom) and six months (top). The blue arrows indicate small crystallised islands. Solid **3** was scattered over the central region of the liquid after four months (seeding).

**Fig. 5:**
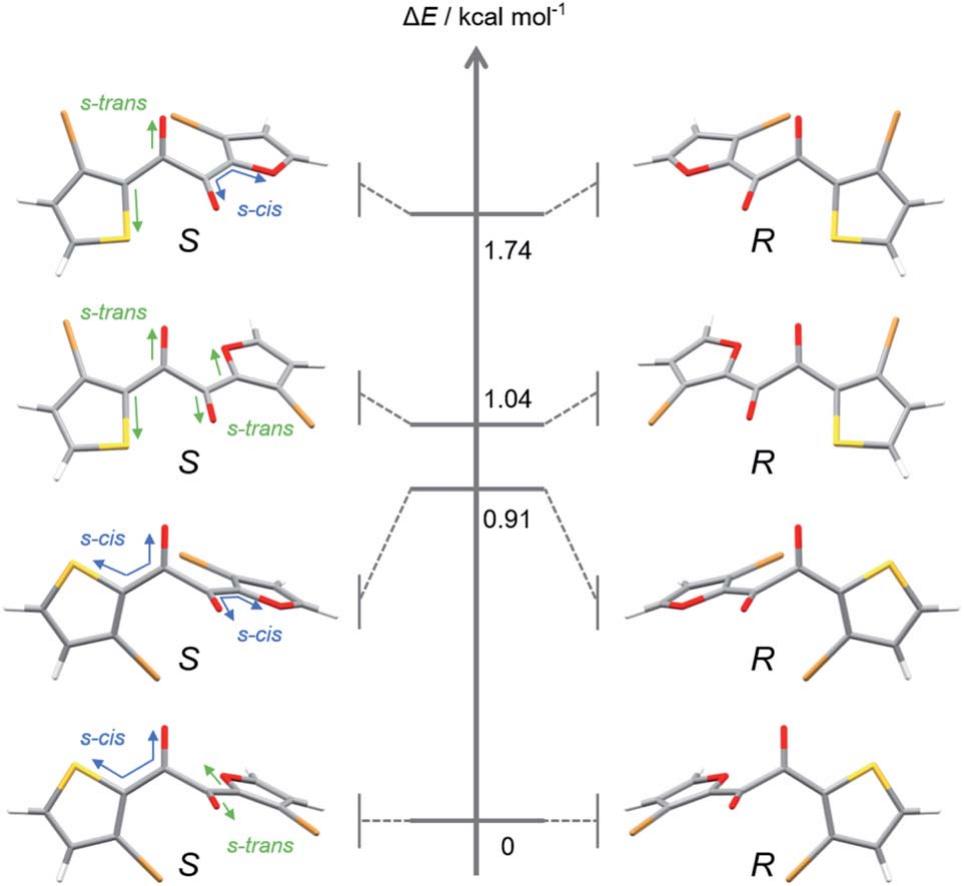
Optimised structures and energy levels of eight conformers of **3′** in the S_0_ state calculated at the B3LYP-D3/6-311G(d) level of theory.

As for kinetic stability (*i.e.*, ease of crystallisation from the SCL),^15*a*^**1** instantaneously crystallises upon evaporation of the solvent, while **2** tends to solidify within a few days at room temperature. In contrast, SCL **3** crystallises significantly slowly. To monitor the solidification process using XRD (Fig. 4), the well of a glass sample holder (20 × 20 × 0.2 mm^3^) was filled with SCL **3** and stored in a container at room temperature in air. At first, the liquid exhibited three broad diffraction peaks near 2*θ* values of 8.6, 14, and 25° (Fig. 4, dark blue pattern). Tiny islands formed after three months, although no sharp peak was detected at that time. Even though solid **3** was further scattered over the liquid after four months (seeding), the crystalline domains propagated slowly, taking six months in total for solidification of more than half of the area (Fig. 4, dark orange pattern and the upper photographic image). Furthermore, the SCL state was stable under mechanical stimulations, such as pricking with a needle (photographic images in Fig. 1c and 2a) or shear stress by a rheometer (high shear frequency up to 16 s^−1^ at 25 °C; Fig. S6†).^18^ Therefore, the SCL state of the unsymmetrical diketone **3** is obviously more stable than those of the symmetrical diketones. The above results demonstrate that desymmetrising a molecular structure is a rational and promising approach to developing materials with highly stable SCL states. Thus, desymmetrisation increases the number of (meta-) stable conformers within a 2 kcal mol^−1^ energy range, from five for the symmetrical core of **1** to eight for the unsymmetrical core of **3**, as confirmed by DFT calculations (Fig. 5, S12, S13, and Table S1†). Moreover, the lack of *C*_2_ symmetry provides an additional degree of freedom in relative molecular orientation. These features efficiently prevent nucleation and growth that form ordered molecular arrangements of crystals, which enhances the kinetic stability of the liquid state.

### Glass transition of the SCL and the thermochromic behaviour

**Fig. 6:**
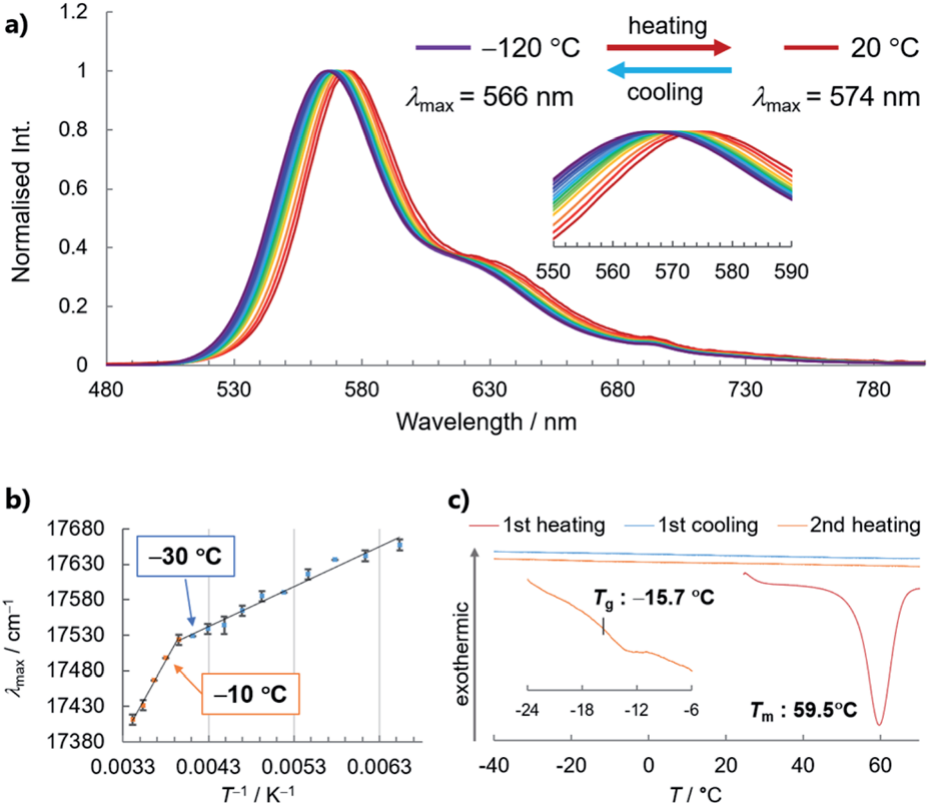
Thermochromic behaviour of liquid **3**. (a) Steady-state PL spectra acquired from −120 to 20 °C in 10 °C steps. (b) Relationship between *λ*_max_ and reciprocal temperature. The error bars were constructed from three independent measurements. (c) DSC thermograms of solid **3** during heating/cooling cycles at 10 °C min^−1^ for the 1^st^ heating and at 0.3 °C min^−1^ for the 1^st^ cooling and 2^nd^ heating processes, under a flow of N_2_.

With a highly stable molecular SCL phosphor in hand, we investigated the temperature-dependence of its optical properties in the SCL and glass states, which revealed a PL spectral shift (*i.e*., thermochromism) that correlates bulk thermal phase behaviour with the microscopic molecular environment (Fig. 6).^8,19^ Steady-state PL spectra were acquired from −120 to 20 °C in 10 °C steps, which showed redshifts in the maximum emission wavelength (*λ*_max_), from 566 nm to 574 nm. The increased intensity at a lower temperature without the emergence of new peaks is indicative of phosphorescence-dominated emission (Fig. S20†). The relationship between *λ*_max_ and reciprocal temperature can be fitted using two lines, with the slope changing between −10 and −30 °C. Note that crystallisation was not observed by differential scanning calorimetry (DSC) even at a very slow cooling/heating rate of 0.3 °C min^−1^, which is another indication of the high stability of the SCL state (Fig. 6c). Instead, DSC revealed that **3** has a glass-to-isotropic liquid-transition temperature (*T*_g_) of −15.7 °C, which is close to where the two lines in Fig. 6b intersect. Therefore, the phosphorescence wavelength of liquid **3** responds to a bulk physical property (such as viscosity) that discontinuously changes at *T*_g_.^20^ The increase in viscosity prevents surrounding molecules from reorganising as the dipole of the excited molecule changes, which we consider to be the reason for SCL/glass **3** exhibiting thermochromic behaviour (Fig. S21†). Although the peak shift is subtle for practical application, to the best of our knowledge, this is the first example of the phosphorescence thermochromism of a single-component organic liquid, whose performance will be improved on the basis of the above working hypothesis.

## Conclusions

Efficient RTP in a solvent-free liquid state in air was realised through desymmetrising a *C*_2_-symmetrical thienyl diketone. The unsymmetrical flexible core kinetically stabilised the SCL state even in the presence of seed crystals or under pricking or shearing stresses. Notably, only the liquid exhibits RTP; the solution and crystalline solid are virtually non-emissive. We suggest that the liquid is sufficiently dense to benefit from the intermolecular (external) heavy atom effect and suppress oxygen diffusion, while being sufficiently flexible to generate an intrinsically emissive TP conformer. In addition, the unsymmetrical diketone exhibits thermochromic phosphorescence, which stems from its fluidity. Our work demonstrates the significance of an unsymmetrical flexible core for realising RTP in a condensed, yet loose liquid state. Investigations into the use of the SCL solid-phase transition with the aim of developing stimuli-responsive RTP is currently ongoing in our laboratory.

## Data availability

All experimental/computational procedures and data related to this article are provided in the ESI.†

## Author contributions

M. K. conceptualisation: equal; data curation: lead; formal analysis: equal; investigation: lead; visualisation: lead; writing—original draft: lead; writing—review & editing: supporting. T. O. conceptualisation: supporting; resources: lead; funding acquisition: supporting; writing—review & editing: supporting. Y. T. conceptualisation: equal; data curation: supporting; formal analysis: equal; funding acquisition: lead; investigation: supporting; visualisation: supporting; writing—original draft: supporting; writing—review & editing: lead.

## Conflicts of interest

There are no conflicts to declare.

## Supplementary Material

SC-012-D1SC03800A-s001

SC-012-D1SC03800A-s002

SC-012-D1SC03800A-s003

SC-012-D1SC03800A-s004
