# Supplementary material for: Room-temperature phosphorescence of a supercooled liquid: kinetic stabilisation by desymmetrisation
Source: Chem Sci. 2021 Sep 10;12(43):14363–8. doi: 10.1039/d1sc03800a (PMC8580009; doi:10.1039/d1sc03800a)
Supplement: SC-012-D1SC03800A-s001 [file SC-012-D1SC03800A-s001.pdf]

Electronic Supplementary Material (ESI) for Chemical Science.
This journal is © The Royal Society of Chemistry 2021

3'_Skew (opt. @S_0)

E(RB3LYP) = –6155.75051019

C	-3.938043057553	-0.325970323411	-1.325167485448
C	-4.395243704499	0.303041658102	-0.206443626604
C	-2.370401949643	-0.370149914487	0.298308424777
C	-2.624398737670	-0.761359112088	-0.997125644542
C	-1.210688851425	-0.440696699010	1.178381171935
O	-1.127903106930	0.148125506771	2.235081863743
C	-0.087363299209	-1.433908295936	0.820677375854
O	-0.287384143785	-2.601413126406	1.098031511837
C	1.196199201698	-0.951464383891	0.315295056360
C	1.591111009211	0.249091033489	-0.240361228066
C	2.947080039169	0.274498812754	-0.655059420353
C	3.571254301035	-0.917563596692	-0.412251272211
S	2.530578431390	-2.085137794368	0.307195642593
Br	0.470719341238	1.755811732747	-0.521237320923
H	-4.453562398557	-0.467105439882	-2.261514172286
H	-5.328991149977	0.785802148050	0.034601051846
H	3.418618661486	1.135770545946	-1.107772690426
H	4.601728091277	-1.169574833620	-0.619234691418
O	-3.470420248258	0.293254941114	0.773864644916
Br	-1.462056868997	-1.649336299182	-2.182222881585

3'_Skew (opt. @T_1)

E(UB3LYP) = –6155.66675402

C	-4.509694449685	-0.656758464500	-0.885795455940
C	-4.625010603548	-0.126185038053	0.363759819561
C	-2.465497553631	-0.323274662391	0.012287651354
C	-3.114014869955	-0.764806212511	-1.130141693889
C	-1.071335715045	-0.114935610666	0.370655599375
O	-0.741596560167	0.886478995493	1.044029392940
C	-0.095576472161	-1.127615102592	-0.041665339178
O	-0.471805758612	-2.276211764814	-0.356419070498
C	1.328190922355	-0.804106314518	0.031359891950

C	2.032253275667	0.317803731010	-0.367589004888
C	3.429189650367	0.226089204854	-0.136609836551
C	3.785613705731	-0.978776443937	0.401695809443
S	2.434526953470	-2.025902344811	0.633888278993
Br	1.296190284182	1.808753581241	-1.278221026478
H	-5.305382531847	-0.928690003442	-1.560798528871
H	-5.470788126801	0.126976658737	0.982530105596
H	4.124347997033	1.019786817325	-0.373270181625
H	4.776686202937	-1.307028751188	0.680159297260
O	-3.408214116352	0.098531515743	0.912877724523
Br	-2.357250673939	-1.278413230982	-2.769687123077

3’_Int$_a$ (opt. @S$_0$)

E(RB3LYP) = –6155.75114629

C	-0.074094397562	-4.376637488039	0.502126342770
C	-0.788022762953	-3.547662172330	-0.318088707331
C	-0.355239649430	-2.201639960863	-0.207405144033
C	0.679941480683	-2.016152181368	0.687884327977
S	1.117125716291	-3.544375104093	1.424482444552
C	1.403726702918	-0.826346545282	1.135051636772
C	1.329194440495	0.478919670578	0.313722125691
O	2.162290706045	-0.838264622886	2.085663049502
C	0.534732721941	1.556733900392	0.878704294324
O	1.990851834380	0.572904734969	-0.698138960974
C	0.283419853033	2.854930362711	0.503188591803
C	-0.645573921092	3.392774175154	1.437449601337
C	-0.901297244496	2.384398329228	2.317897294752
O	-0.204578933305	1.273411580227	2.001169779684
Br	1.002323151005	3.770568051029	-0.968529059813
Br	-1.219252086305	-0.830698135208	-1.198782245787
H	-0.188598633624	-5.445980265898	0.610057358902
H	-1.586208176540	-3.867751263264	-0.973487540704
H	-1.055641113115	4.390060161405	1.442113493162
H	-1.530708689764	2.311240243684	3.190312839787

3'_Int$_a$ (opt. @T$_1$)

E(UB3LYP) = –6155.66946694

C	0.677453041507	-4.536990430427	0.104379224668
C	-0.248938232861	-3.956384666648	-0.715709471393
C	-0.515004456693	-2.609836957601	-0.355818484290
C	0.243344044567	-2.160619709725	0.709644275126
S	1.243225442369	-3.461865797456	1.329954783783
C	0.263799039865	-0.862022307006	1.395637901452
C	0.418165780665	0.338448439673	0.537100184588
O	0.230972235796	-0.825280179104	2.637695568755
C	0.229507351847	1.657771061204	1.087999048913
O	0.711661956843	0.169184997824	-0.668430243002
C	0.342237329797	2.905501720014	0.507770354090
C	0.028428297165	3.865040038466	1.510261975044
C	-0.260053098028	3.142612419119	2.627562524978
O	-0.147342808607	1.813173498258	2.391052066230
Br	0.827980854168	3.290875645394	-1.265543000172
Br	-1.892469926571	-1.627064182706	-1.209017492598
H	1.065807188848	-5.543802492257	0.049489040704
H	-0.737101119001	-4.459135924648	-1.539167686542
H	0.021439658693	4.937686517152	1.400402843408
H	-0.548721581761	3.413141780617	3.630128108629

3'_Int$_b$ (opt. @S$_0$)

E(RB3LYP) = –6155.74807639

C	-4.387910493840	-1.312776127287	-0.669203357196
C	-4.986054450095	-0.259957759986	-0.045345713775
C	-2.838267856516	-0.006805370284	0.324822329136
C	-2.995253915419	-1.149858357150	-0.430503704523
C	-1.699181703913	0.765992779578	0.810877389115
O	-1.790893626279	1.926699908432	1.164606409600
C	-0.358883498852	0.027770130436	0.983634999198
O	-0.359168597587	-0.996933449737	1.633901645598
C	0.839563421943	0.641934097341	0.405133696457

C	2.091443825353	0.074830734423	0.238310061223
C	3.016179400130	0.905590269964	-0.441650512654
C	2.463494908439	2.108073820553	-0.785716576883
S	0.824250557171	2.254929197954	-0.288545636411
Br	2.599415919481	-1.663884631756	0.792864322654
H	-4.859841753061	-2.104668496092	-1.228333871726
H	-6.016202723299	0.039253484233	0.065913811147
H	4.034938151971	0.613538680926	-0.655708872855
H	2.948095361698	2.931929697849	-1.290480366839
O	-4.076920162708	0.539276778358	0.545032280241
Br	-1.666243484619	-2.283346607756	-1.131331221509

3’_Int$_b$ (opt. @T$_1$)

E(UB3LYP) = –6155.67211059

C	-4.611150845285	-1.473410417267	-0.218056862517
C	-5.013083388463	-0.348521036132	0.434110446457
C	-2.870246977371	-0.062504140628	0.047333467228
C	-3.227145489978	-1.285199858526	-0.490559742425
C	-1.651759270192	0.750608187800	0.032291178886
O	-1.706512946142	1.967422593790	-0.198672930563
C	-0.384694290463	0.054108089156	0.330036413990
O	-0.467631651296	-1.124863589756	0.780116089174
C	0.878977076187	0.712351412157	0.118914280130
C	2.141360720587	0.166359743353	0.306213872233
C	3.206110782354	1.047023139703	0.003239900290
C	2.749871930166	2.265318140188	-0.415535989306
S	1.024179207219	2.371166508685	-0.449296488501
Br	2.464872300707	-1.600874460701	0.914416202221
H	-5.210794399645	-2.327618699748	-0.489002641825
H	-5.956062271518	-0.033316425117	0.850847749988
H	4.249650247890	0.778904773559	0.095723171737
H	3.338543323642	3.124959283990	-0.702815049568
O	-3.984837121133	0.519829498173	0.586549497608
Br	-2.187087657266	-2.450153962678	-1.537575455238

3'_TP (opt. @S_0)

E(RB3LYP) = –6155.74960051

C	-4.981138751883	-0.452081814782	-0.430982900440
C	-4.072446493264	-1.403475597151	-0.789401256930
C	-2.899609625579	0.191891446760	0.154160202866
C	-4.221642849030	0.584895542655	0.174743839686
C	-1.676987756349	0.851696842409	0.589607920899
O	-1.662243503978	2.004438185478	0.983891321513
C	-0.380039211914	0.011910497044	0.611664584542
O	-0.431982140711	-1.128491914793	1.022727925502
C	0.859489824361	0.672812761630	0.179682749587
C	2.093577534933	0.085573224405	-0.050331040415
C	3.081056344581	0.979677330285	-0.531823628120
C	2.595683468863	2.251185158301	-0.661792842359
S	0.946582061178	2.387670744322	-0.195822065322
Br	2.517377158384	-1.745854800641	0.192230573479
H	-6.049632882652	-0.473775084475	-0.573495194671
H	-4.164165640635	-2.360318887885	-1.278320797324
H	4.093741767520	0.680207677463	-0.763646405601
H	3.136271924167	3.128228255466	-0.989261909028
O	-2.822790608471	-1.039068992981	-0.448069026210
Br	-4.937907599520	2.174636566491	0.869203658349

3'_TP (opt. @T_1)

E(UB3LYP) = –6155.67648896

C	-5.096967450498	-0.509866314521	-0.028652259959
C	-4.287513125031	-1.595453454337	-0.164640751970
C	-2.932919303591	0.128733012578	-0.046916391618
C	-4.223421637578	0.610974218091	0.048197405967
C	-1.661490194051	0.819990201840	-0.026408340041
O	-1.598490698523	2.051696346717	0.091568068793
C	-0.386474440207	0.028590622657	-0.150932965384
O	-0.436911116534	-1.225066707891	-0.271475047520
C	0.873087305571	0.727481350411	-0.129370318227

C	2.132441428248	0.145774555682	-0.232825621939
C	3.204613930181	1.065193814839	-0.183044630905
C	2.760028559203	2.349684495914	-0.041245330494
S	1.036055058833	2.472753613023	0.034875796766
Br	2.444925008249	-1.715557918538	-0.425994212395
H	-6.174331594890	-0.493388155445	0.013073415173
H	-4.474173494375	-2.652588722432	-0.260871078430
H	4.244618129736	0.776337092803	-0.249800252132
H	3.356521356734	3.248679416546	0.024247949547
O	-2.983654326008	-1.228717137675	-0.177358916427
Br	-4.772750375470	2.396506809738	0.242539191193
