# Supplementary material for: Room-temperature phosphorescence of a supercooled liquid: kinetic stabilisation by desymmetrisation
Source: Chem Sci. 2021 Sep 10;12(43):14363–8. doi: 10.1039/d1sc03800a (PMC8580009; doi:10.1039/d1sc03800a)
Supplement: SC-012-D1SC03800A-s002 [file SC-012-D1SC03800A-s002.pdf]

Electronic Supplementary Material (ESI) for Chemical Science.
This journal is © The Royal Society of Chemistry 2021

Electronic Supplementary Information

for

Room-temperature phosphorescence of a supercooled liquid: kinetic stabilisation by desymmetrisation

Mao Komura, Takuji Ogawa, and Yosuke Tani*

Department of Chemistry, Graduate School of Science, Osaka University
1-1 Machikaneyama, Toyonaka, Osaka 560-0043, Japan
E-mail: y-tani@chem.sci.osaka-u.ac.jp

Table of Contents

1. Instrumentation and chemicals 2
2. Synthesis procedures 3
3. Optical, photophysical, and rheological properties of liquid **3** 6
4. Photophysical properties in solution 10
5. Density functional theory calculations 15
6. Photophysical and structural properties of solid **3** 18
7. Thermal property and thermochromic behavior of **3** 22
8. NMR charts 26
9. References 34

1. Instrumentation and chemicals

Unless otherwise noted, all the reactions were performed under a nitrogen atmosphere using anhydrous solvents and heat-gun-dried glassware on a dual-manifold Schlenk line. ^{1}H and $^{13}C\{^{1}H\}$ NMR spectra were recorded on a JEOL ECS400 spectrometer. Chemical shift values (δ) are reported in ppm and are calibrated to residual solvent (7.26 ppm in $CDCl_3$) for ^{1}H and to $CDCl_3$ (77.0 ppm) or tetramethylsilane (0.00 ppm) for ^{13}C NMR. Melting points were measured using a Hitachi NEXTA DSC200 or a Rigaku Thermo Plus EVO II under a flow of nitrogen at a heating rate of 10 °C min^{-1}. High-resolution mass spectra (ESI-HRMS) were obtained with a Thermo Fisher Scientific LTQ Orbitrap XL mass spectrometer. Elemental analysis (EA) was conducted on a Yanaco MT-6 recorder.

Analytical thin-layer chromatography (TLC) was performed on aluminum plates bearing a layer of Merck silica gel 60 F_{254}. Column chromatography was carried out on silica-gel 60 (Kanto Chemical Co., Inc., spherical, 63–210 μm). Gel permeation chromatography (GPC) was performed using a JAI LC-9130 NEXT equipped with JAIGEL-1HR and 2HR (eluent: $CHCl_3$, flow rate: 10 mL/min).

Anhydrous tetrahydrofuran (THF) and CH_2Cl_2 were purchased from Wako Chemical and Kanto Chemical, respectively, and further purified by passage through activated alumina under positive nitrogen pressure as described by Grubbs et al.[1] Unless otherwise noted, chemicals obtained from commercial suppliers were used without further purification. 2-(Pentafluorophenyl)-6,7-dihydro-5*H*-pyrrolo[2,1-*c*][1,2,4]triazol-2-ium tetrafluoroborate (**S2**), 1,2-bis[3-bromo-5-(triisopropylsilyl)thiophen-2-yl]ethane-1,2-dione (**1**), and 1,2-bis[5-(triisopropylsilyl)furan-2-yl]ethane-1,2-dione (**2**) were synthesized according to the literatures.[2]

All the photophysical properties (*i.e.*, UV-vis absorption, photoluminescence (PL), and excitation spectra, PL quantum yields (PLQYs), and PL lifetimes) were obtained at room temperature (RT) under air unless otherwise noted. UV-vis absorption and PL spectra of solutions were acquired using a Shimadzu UV-3150 spectrometer and a JASCO FP-8200 spectrofluorometer, respectively. Solid- and liquid-state PL spectra were obtained using a JASCO FP-8200 spectrofluorometer. PLQY of a solution of **3** was determined by the relative method using quinine sulfate as a standard[3] using a JASCO FP-8200 spectrometer. PLQYs of **3** liquid and solid were determined by the absolute method using a Hamamatsu photonics C9920-02 spectrometer with an integrating sphere. PL lifetime measurements for liquid, solid, and a solution were performed using a HORIBA DeltaFlex multichannel scaling system using DeltaDiode for excitation (368 nm). Powder X-ray diffraction (PXRD) patterns were collected on a Rigaku MiniFlex600 with CuKα radiation (λ = 1.5418 Å) using D/teX Ultra as a detector.

2. Synthesis procedures

Synthesis of 3

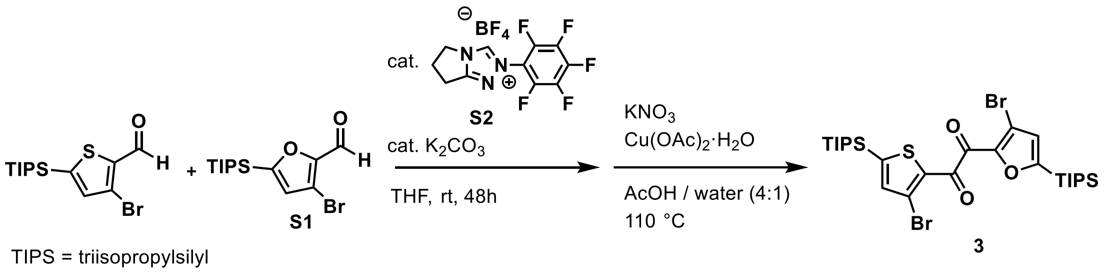

Scheme S1. Synthesis of **3**

3-Bromo-5-(triisopropylsilyl)furan-2-carbaldehyde (S1)

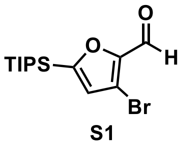

Aldehyde **S1** was synthesized according to a literature with a modified procedure.[4] To a 50 mL 2-neck flask were added diisopropylamine (2.1 mL, 15 mmol) and anhydrous THF (7.5 mL), and cooled to −30 °C. To the stirred mixture was added *n*-butyllithium (BuLi, 2.65 M in hexane, 4.5 mL, 12 mmol), and the stirring was continued for 10 min at –30 °C and 10 min at 0 °C. To the mixture was added a solution of potassium *tert*-butoxide (1.12 g, 10 mmol) in THF (16 mL), and then the resulting mixture was cooled to −78 °C and stirred for 10 min. 2-Bromo-5-(triisopropylsilyl)furan (3.05 g, 10 mmol), synthesized according to a literature,[2,5] was dissolved in anhydrous THF (10 mL) and was added to the mixture. The temperature was reached to –58 °C while stirring for 1 h, during which the solution color turned to slight orange. Then, to the solution was added *N*,*N*-dimethylformamide (1.2 mL, 15 mmol) dropwise. The reaction mixture was warmed up to RT, stirred for 15 minutes, and then quenched by adding water and extracted with Et_2O three times. The combined organic layers were dried over $MgSO_4$, filtered, and then evaporated. The residue was purified by silica-gel column chromatography (hexane/CH_2Cl_2 = 3:1) to give **S1** (1.743 g, 53%) as a light-yellow liquid. **^{1}H NMR** (400 MHz, $CDCl_3$) δ: 9.75 (s, 1H), 6.83 (s, 1H), 1.40-1.33 (m, 3H), 1.12 (d, *J* = 7.2 Hz, 18H). **^{13}C NMR** (100 MHz, $CDCl_3$) δ: 175.96, 166.22, 151.37, 126.52, 111.92, 18.27, 10.72. **ESI-HRMS** (*m*/*z*): $[M+Na]^+$ calcd for $C_{14}H_{23}BrO_2SiNa$, 353.0543; found, 353.0511.

1-(3-Bromo-5-(triisopropylsilyl)furan-2-yl)-2-(3-bromo-5-(triisopropylsilyl)thiophen-2-yl)ethane-1,2-dione (3)

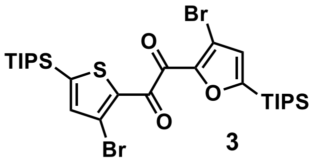

Unsymmetrical diketone **3** was synthesized via the cross-benzoin condensation and oxidation with a modified procedure.[2,6] To a Schlenk tube were added K_2CO_3 (6.0 mg, 0.04 mmol) and triazolium salt **S2** (15.3 mg, 0.04 mmol), and the tube was shortly evacuated and backfilled with N_2 three times. THF (1.0 mL) was added via a syringe and the suspension was stirred for 15 minutes. To the pale red suspension were added **S1** (0.35 g, 1.06 mmol) and 3-bromo-2-formyl-5-(triisopropylsilyl)thiophene[2] (0.791 g, 2.11 mmol) in THF (0.5 mL) under a flow of N_2. The mixture was stirred for 48 h at RT, and then quenched by adding water. The organic layer was separated and the aqueous layer was extracted with Et_2O. The combined organic extracts were dried over $MgSO_4$, filtered, and then evaporated. The residue was purified by silica-gel column chromatography (hexane/CH_2Cl_2 = 1:1). Then, to a Schlenk tube were added the obtained benzoins, KNO_3 (134 mg, 1.32 mmol), $Cu(OAc)_2{\cdot}H_2O$ (15 mg, 0.074 mol), and AcOH/H_2O (4:1, 5.0 mL). The mixture was stirred for 2 h at 110 °C. After cooling to RT, the reaction was quenched by adding H_2O and CH_2Cl_2. The organic layer was separated and the aqueous layer was extracted with CH_2Cl_2 three times. The combined organic extracts were dried over $MgSO_4$, filtered, and then evaporated. The residue was purified by silica-gel column chromatography (hexane/CH_2Cl_2 = 2:1). Evaporation of the solvents afforded **3** (397 mg, 55%) as an orange oil. The oil was eventually solidified to a white powder. **m.p.** 59.5 °C. **^{1}H NMR** (400 MHz, $CDCl_3$) δ: 7.20 (s, 1H), 6.87 (s, 1H), 1.40-1.33 (m, 3H), 1.28-1.21 (m, 3H), 1.11 (d, *J* = 7.2 Hz, 18H), 1.03 (d, *J* = 7.2 Hz, 18H). **^{13}C NMR** (100 MHz, $CDCl_3$) δ: 184.07, 179.89, 167.01, 149.71, 148.61, 140.27, 138.78, 127.73, 118.36, 110.79, 18.34, 18.27, 11.50, 10.73. **EA** Calcd for $C_{28}H_{44}Br_2O_3SSi_2$: C, 49.70; H, 6.55. Found: C, 49.79; H, 6.58.

Synthesis of 2

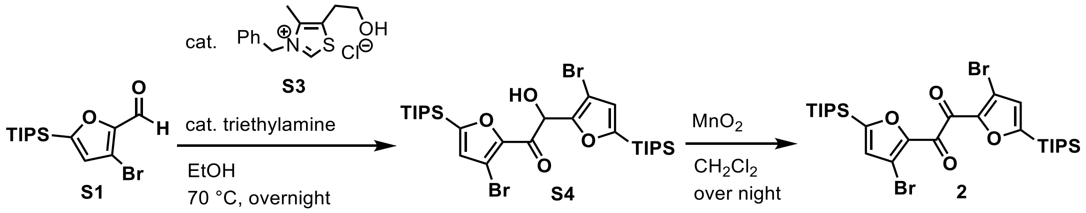

Scheme S2. Synthesis of **2**

1,2-Bis(3-bromo-5-(triisopropylsilyl)furan-2-yl)-2-hydroxyethan-1-one (S4)

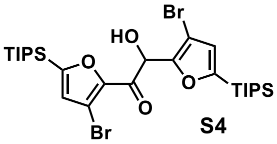

S4

To a Schlenk tube were added **S1** (0.331 g, 1.0 mmol), thiazolium salt **S3** (14.3 mg, 0.05 mmol), and EtOH (1.0 mL). The tube was shortly evacuated and backfilled with N_2 three times, after which triethylamine (10 μL, 0.07 mmol) was added. The mixture was stirred at 70 °C overnight, and then quenched at RT by adding sat. NH_4Cl aq. The organic layer was separated and the aqueous layer was extracted with CH_2Cl_2 three times. The combined organic extracts were dried over $MgSO_4$, filtered, and then evaporated. The residue was purified by silica-gel column chromatography (hexane/CH_2Cl_2, 1:1) to give **S4** (0.24 g, 72%) as a colorless oil. **^{1}H NMR** (400 MHz, $CDCl_3$) δ: 6.77 (s, 1H), 6.59 (s, 1H), 5.91 (d, *J* = 4.8 Hz, 1H), 4.31 (d, *J* = 5.5 Hz, 1H), 1.39-1.24 (m, 3H), 1.22-1.08 (m, 3H), 1.02-0.96 (m, 36H). **^{13}C NMR** (100 MHz, $CDCl_3$) δ: 183.71, 165.32, 158.75, 151.99, 149.12, 127.89, 125.14, 110.26, 100.43, 67.13, 18.33, 18.27, 18.25, 10.68. Only one resonance peak for the methine carbons $SiCH(CH_3)_2$ was observed owing to overlapping. **ESI-HRMS** (*m*/*z*): $[M+Na]^+$ calcd for $C_{28}H_{46}Br_2O_4Si_2Na$, 685.1173; found, 685.1253.

1,2-Bis(3-bromo-5-(triisopropylsilyl)furan-2-yl)ethane-1,2-dione (2)

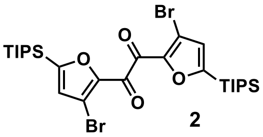

2

The diketone **2** was synthesized according to a literature.[7] To a 50 mL flask were added **S5** (238 mg, 0.714 mmol), MnO_2 (621 mg, 7.14 mmol), and dry CH_2Cl_2 (7 mL). The mixture was stirred overnight at RT. The mixture was filtered through a pad of Celite, and then evaporated. The residue was purified by silica-gel column chromatography (hexane/AcOEt, 20:1) and GPC to give **2** (70 mg, 15%) as a slight brown liquid, which was solidified upon standing to a white solid. **m.p.** 60.6 °C. **^{1}H NMR** (400 MHz, $CDCl_3$) δ: 6.84 (s, 2H), 1.27–1.24 (m, 6H), 1.04 (d, *J* = 7.6 Hz, 36H). **^{13}C NMR** (100 MHz, $CDCl_3$) δ: 179.95, 167.23, 149.54, 127.63, 110.90, 18.29, 10.77. **EA** Calcd for $C_{28}H_{44}BrO_4Si_2$: C, 50.91; H, 6.71. Found: C, 50.82; H, 6.67.

3. Optical, photophysical, and rheological properties of liquid 3

Optical inspection

Solvent-free liquid **3** was inspected under an Olympus BX60M polarized and fluorescence microscope. Photographic images in Figure S1 were taken using SONY NEX-5N.

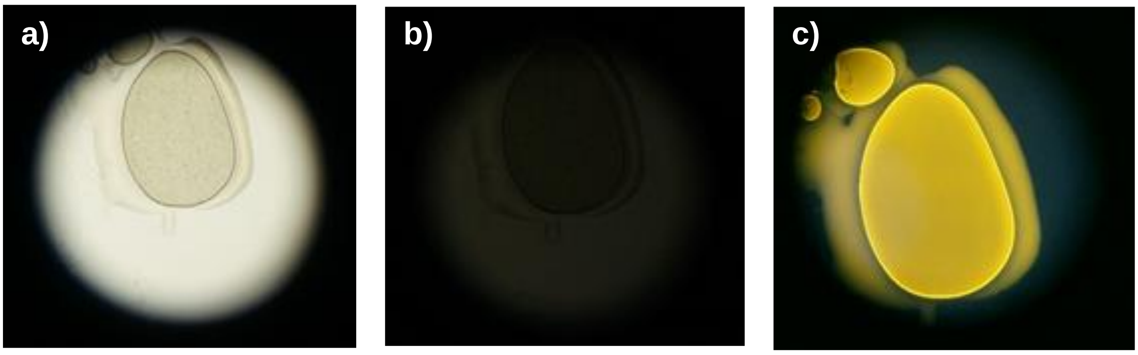

Figure S1. Polarizing optical microscope images of liquid **3** with a) open Nichol prisms and b) crossed Nichol prisms. c) A fluorescence microscope image taken through U-MWUS mirror unit (Olympus, excitation; 330–385 nm, emission; >420 nm).

Purity of the measurement sample

The purity of **3** was evaluated by recycling size-exclusion chromatography using a JAI LC-9130 NEXT instrument equipped with JAIGEL-1HR and 2HR (eluent: $CHCl_3$, flow rate: 10 mL/min) with a UV detector (254 nm). No trace of any impurity was detected (Figure S2). In addition, before and after recrystallization from MeOH, the photoluminescence spectra (acquired as the SCL after the crystal had been melted) were identical (Figure S3) and the lifetimes were almost identical (16 vs 15 μs, Figure S4). The spectra of liquid **3** from different synthesis batches were also identical (Figure S3).

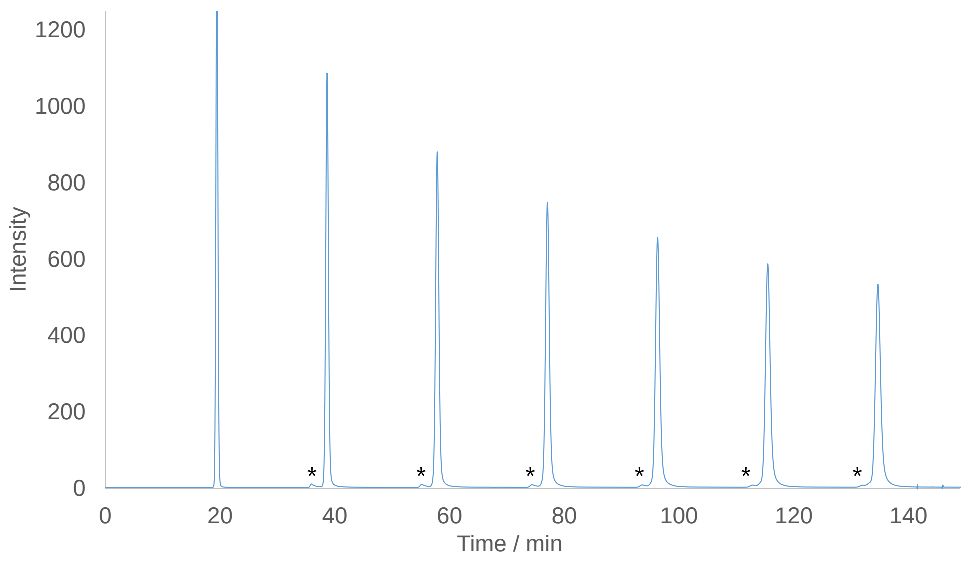

Figure S2. Recycling size-exclusion chromatogram of **3**. Asterisks indicate recycling artifacts.

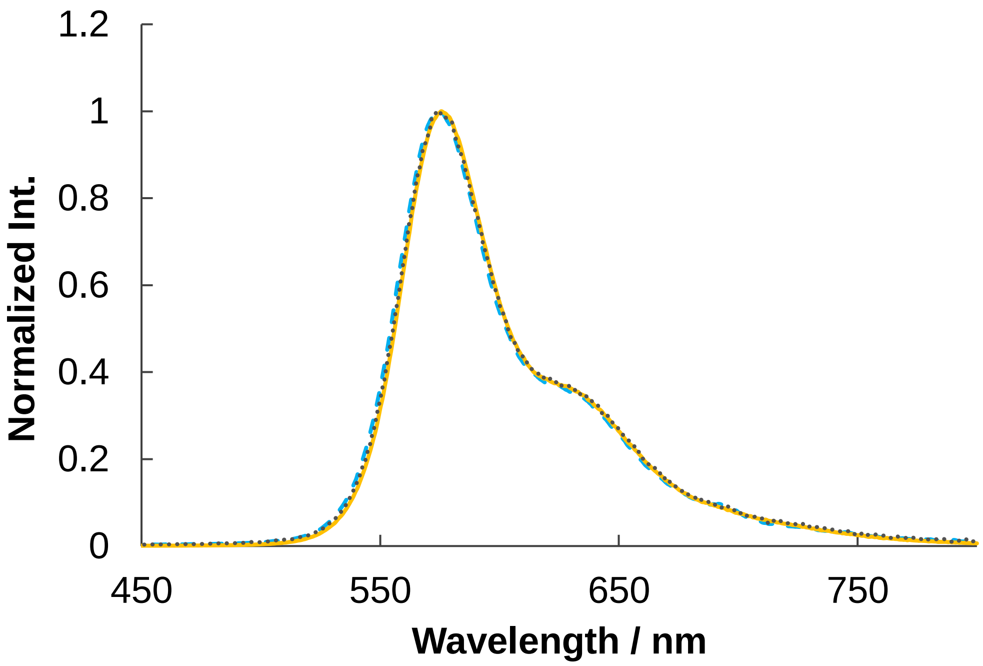

Figure S3. Photoluminescence spectra of liquid **3** before (blue broken line) and after recrystallization from MeOH (yellow solid line), and from a different synthesis batch (grey dotted trace) at RT in air.

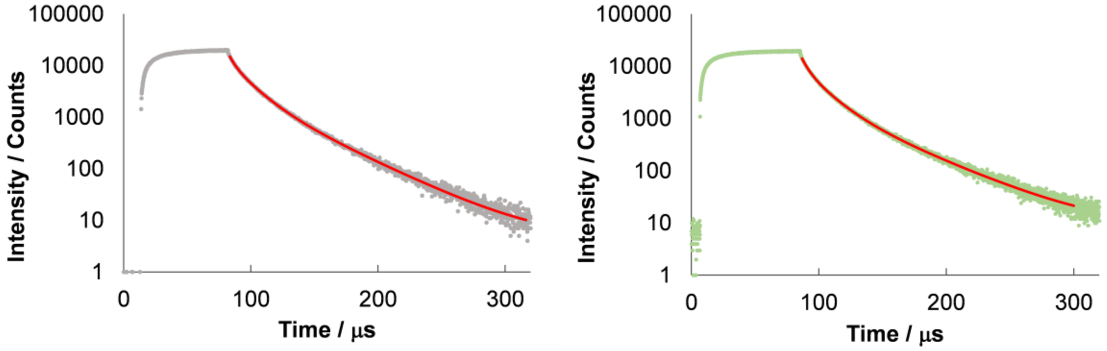

Figure S4. Photoluminescence decay curves of liquid **3** before (left) and after recrystallization from MeOH (right) at RT in air. The red lines are fitted curves. PL intensities were recorded at 570 nm (λ_{ex} = 368 nm).

PL spectra of 3 in the solvent-free liquid state

Liquid **3** was placed in a quartz tube under air or N_2. The PL spectrum was obtained (λ_{ex} = 368 nm) using a JASCO FP-8200 spectrometer quipped with a CoolSpeK USP-203-B cryostat (UNISOKU) with a L42 sharp cut filter (HOYA, long pass, >420 nm) and a U340 band pass filter (HOYA). The spectra matched well with each other when normalized, which indicate the phosphorescence-dominant character of the emission.

PLQY of 3 in the solvent-free liquid state

PLQY of liquid **3** was determined to be 1.0% as an average of four measurements using a Hamamatsu photonics C9920-02 with an integrating sphere (λ_{ex} = 368 nm). For each measurement, the sufficient absorption of the sample (46–60% of the excitation photons) was confirmed. The λ_{ex} different from the absorption maximum was chosen because both the PLQY and PL lifetime must be determined with the same λ_{ex} to derive valid k_p.

PL lifetime of 3 in the solvent-free liquid state (Figure 2b)

The PL decay curve of liquid **3** was acquired with a HORIBA DeltaFlex multichannel scaling system using DeltaDiode for excitation (368 nm). The area-weighted average lifetime τ_p = 16 μs was obtained from a triple-exponential fit using a HORIBA EZTime software (τ_1 = 14 μs (53%), τ_2 = 4.2 μs (28%), τ_3 = 37 μs (19%)). The reduced chi-square of the curve fitting was 1.06. PL intensity was recorded at 570 nm.

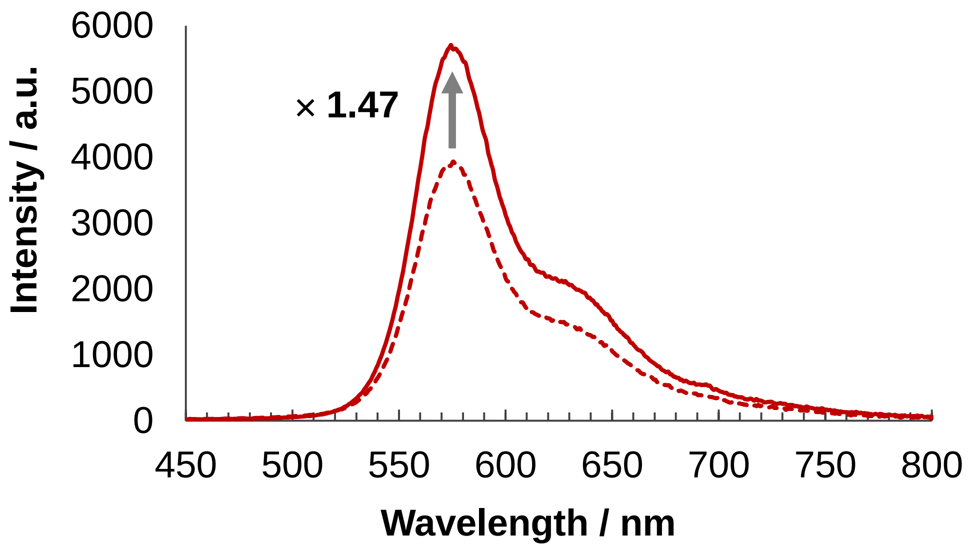

Figure S5. Steady-state PL spectra of liquid **3** (dash line: in air, solid line: in N_2 atmosphere, λ_{ex} = 368 nm).

Rheology experiment

Rheology experiments were carried out using an ARES G2 (TA Instruments Inc.) rheometer. Liquid **3** was sandwiched between two parallel plates so that it completely filled a gap of 1.0 mm. The complex shear modulus $G^* = G' + iG''$, where G' is the storage modulus and G'' is the loss modulus, were measured at 25 °C in an angular frequency ω range from 1 to 100 rad·s^{-1} under oscillatory shear deformation (γ= 1%). The complex viscosity $|\eta^*| = (\eta'^2 + \eta''^2)^{1/2}$, where $\eta' = G''/\omega$ and $\eta'' = G'/\omega$, matched well with η' (Figure S6a). Therefore, the zero-shear viscosity η_0 of liquid **3** was determined to be 45 ± 2 Pa·s as an average of η' in the angular frequency range from 1 to 100 rad·s^{-1} (shear stress from 46 to 2615 Pa). After the experiments, the sample was heated up to ca. 50 °C (below T_m, 59.5 °C), and then the upper plate was retracted. However, the sample remained as liquid (Figure S6b). For comparison, Kim *et al.* reported that shear frequency of 0.03 s^{-1} induced crystallization of stable supercooled molecular liquid (reference 18 in the main text, J. Kim *et al.*, *ACS Cent. Sci.* 2015). Note that angular frequency of 1 and 100 rad·s^{-1} corresponds to frequency of 0.16 and 16 s^{-1}, respectively.

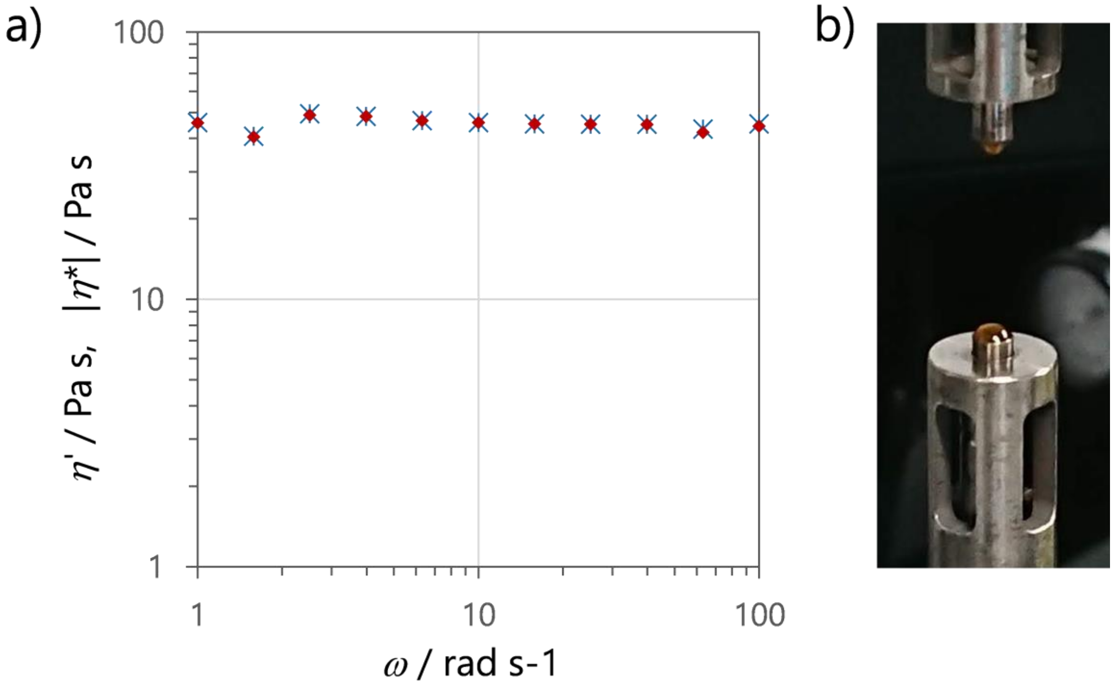

Figure S6. a) Variation of complex viscosity $|\eta^*|$ (blue asterisk) and its real part η' (red diamond) as a function of angular frequency ω for liquid **3** under oscillatory shear deformation $\gamma = 1\%$ at 25 °C. b) A photographic image of liquid **3** after the rheology experiments.

4. Photophysical properties in solution

PL, excitation, and UV-vis absorption spectra of 3 in cyclohexane

Analytically pure **3** was dissolved in spectral-grade cyclohexane (2.2×10^{-4} M), and degassed by Ar bubbling for 10 min. The PL spectrum was obtained (λ_{ex} = 368 nm) using a JASCO FP-8200 spectrometer. The excitation (λ_{em} = 570 nm) and UV-vis absorption spectra were obtained for a diluted solution (2.2×10^{-5} M) using the JASCO FP-8200 spectrometer and a Shimadzu UV-3150 spectrometer, respectively.

The PL spectrum almost overlapped that of **3** in solvent-free liquid state (Figure S7), suggesting the conformational similarity of the emitting species. According to our previous study,[2] in solution, **3** exists mainly as a skew conformer and relax to a planar conformer after photoexcitation, which is common behavior to aromatic 1,2-diketones.[8] Therefore, we consider that the emissive conformer of **3** in solvent-free liquid state is also the planar conformer.

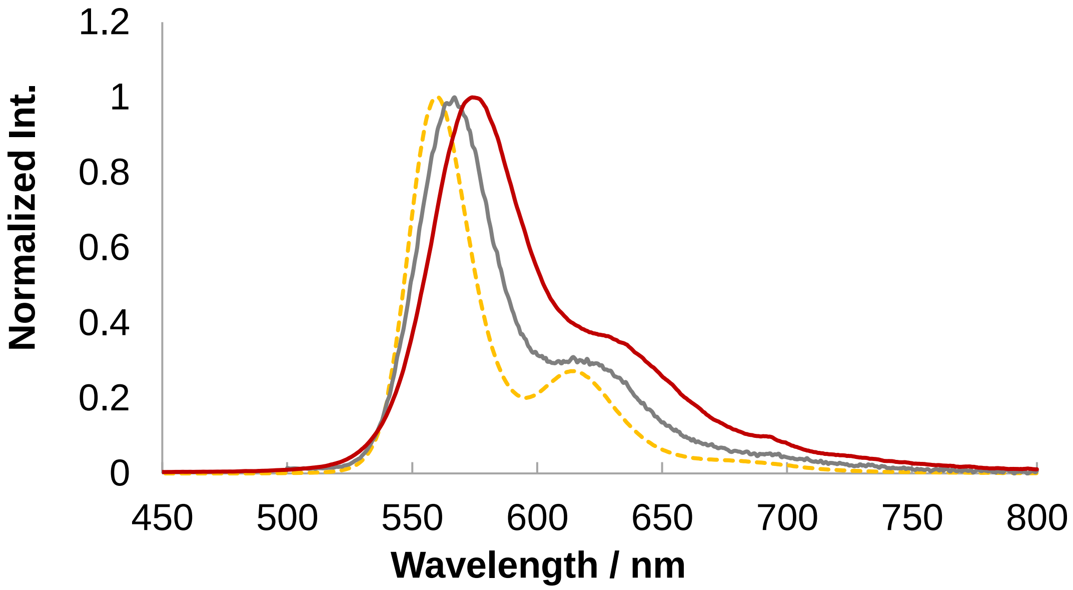

Figure S7. Normalized steady-state PL spectra of **3** in cyclohexane (gray solid line, under Ar), **3** liquid (red solid line, under air), and **1** in cyclohexane (yellow broken line, under Ar). λ_{ex} = 368 nm.

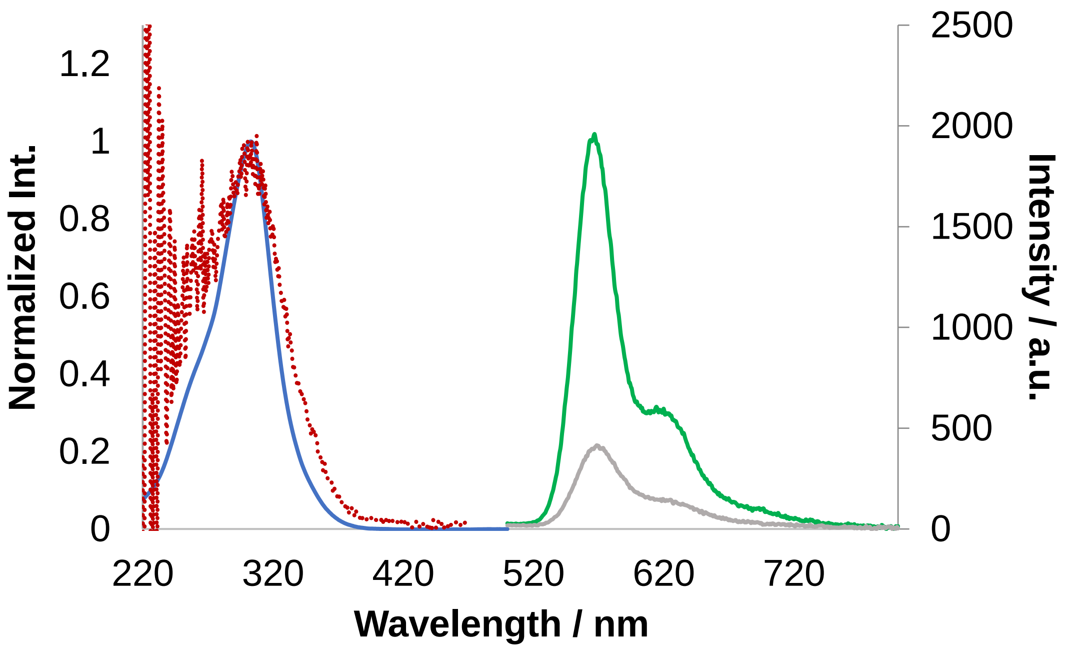

Figure S8. Absorption (blue solid line), excitation (red dot line, λ_{em} = 570 nm), and steady-state PL spectra (green solid line; Ar bubbling for 10 min, gray solid line; Air bubbling) of **3** in cyclohexane (2.2×10^{-5} M for the absorption and excitation spectra, and 2.2×10^{-4} M for the PL spectrum).

Comparison of the absorption spectra of 3 in cyclohexane and liquid

UV-vis absorption spectrum of **3** in solvent-free liquid state was obtained using a Shimadzu UV-3150 spectrometer with a Shimadzu ISR-3100 integrating sphere attachment. Liquid sample was prepared as a thin film by drop-casting onto a quartz substrate. The spectrum overlapped with that of **3** in cyclohexane, indicating the conformational similarity in the ground state (S_0).

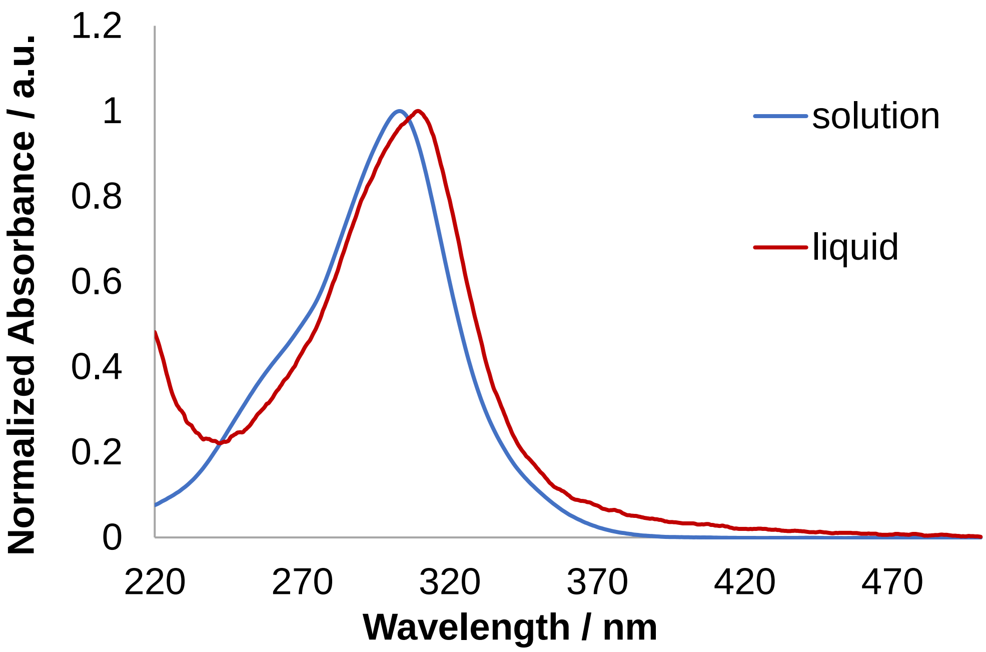

Figure S9. Normalized absorption spectra of **3** in cyclohexane (blue) and liquid (red).

PL lifetime of 3 in solution

The PL decay curve of **3** in the degassed cyclohexane solution was acquired immediately after the measurement of the PL spectrum in Figure S8 with a HORIBA DeltaFlex multichannel scaling system using DeltaDiode for excitation (368 nm). The area-weighted average lifetime τ_p = 33 μs was obtained from a double-exponential fit using a HORIBA EZTime software (τ_1 = 11 μs (39%), τ_2 = 47 μs (61%)). The reduced chi-square of the curve fitting was 1.04. PL intensity of **3** was recorded at 570 nm.

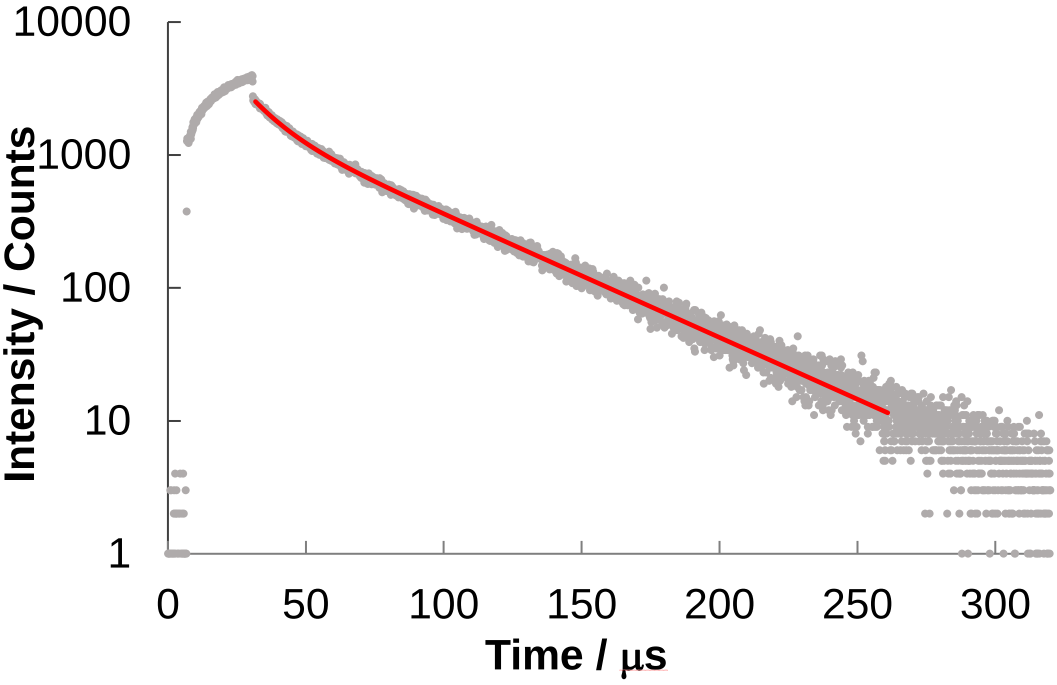

Figure S10. PL decay curve of **3** in cyclohexane (2.2×10^{-4} M) at RT under Ar. The red line denotes the fit to the curve. The PL intensity at 570 nm was recorded (λ_{ex} = 368 nm).

External heavy atom effect in solution

To investigate the external heavy atom effect on the RTP of **3**, PL decay curves were acquired in degassed cyclohexane and ethyl iodide (5:1 and 11:1) solvent mixtures using a HORIBA DeltaFlex multichannel scaling system and a DeltaDiode for excitation (368 nm), with PL intensity recorded at 570 nm. The area-weighted average lifetime was confirmed to shorten and approach that of the solvent-free liquid (16 μs) with increasing ethyl iodide proportion: 33, 27, and 22 μs for 1:0, 11:1, and 5:1 cyclohexane/ethyl iodide, respectively (Figure S11). These results suggest that the external heavy atom effect of ethyl iodide provides a similar outcome to the solvent-free liquid state by increasing the rates of spin-forbidden processes.

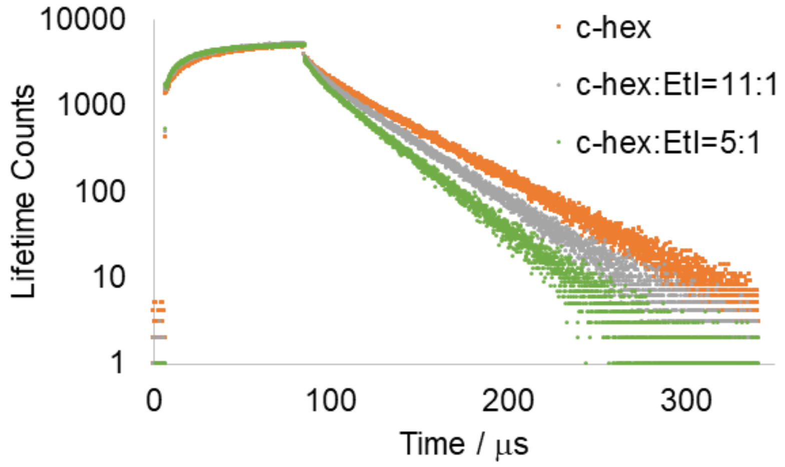

Figure S11. Photoluminescence decay curves of **3** in 1:0, 11:1, and 5:1 cyclohexane/ethyl iodide (2.2×10^{-4} M) at RT under Ar.

5. Density functional theory calculations

(Time dependent (TD)-)density functional theory (DFT) calculations were conducted at the (U)B3LYP-D3/6-311G(d) level of theory using Gaussian 16 program package.[8] According to our previous research, the four conformers of (*S*)-**3'**—Skew, Int_a, Int_b, and TP—were optimized both in the ground state and the T_1 state. Frequency calculation confirmed that all the optimized structures have no imaginary frequency. **3'**-TP (C_s symmetry, Fig. 2c in the main text) is the most stable conformer in the T_1 states. In addition, starting from the (*S*)-**3'**-Int_a in the ground state, we scanned the coordinates around each one of the two thiophene–carbonyl bonds. The step of the scan was set to 20°. At each structure, single-point TD-DFT calculations were performed to obtain the S_1 and T_1 energy levels. From the results, we concluded that **3'**-TP (Fig. 2c in the main text) is accessible upon photoexcitation of **3'**-Int_a, which is the same conformer as that found in the crystal structure.

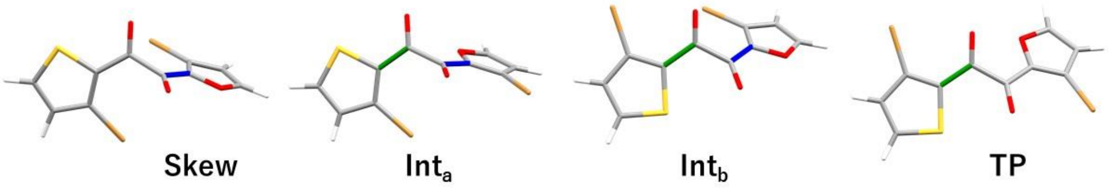

Figure S12. Optimized structures of (*S*)-**3'** in the ground state.

Table S1. The relative Gibbs free energies (ΔG) of four conformers of **3'** and the corresponding dihedral angles (θ) of the two carbonyls. The energies include thermal correction at 298.150 K.

	S_0 opt		T_1 opt	
	ΔG / kcal mol^{-1}	θ / °	ΔG / kcal mol^{-1}	θ / °
**Skew**	0.91	95	5.60	159
**Int_a**	0	97	3.87	166
**Int_b**	1.74	123	2.64	168
**TP**	1.04	135	0	180

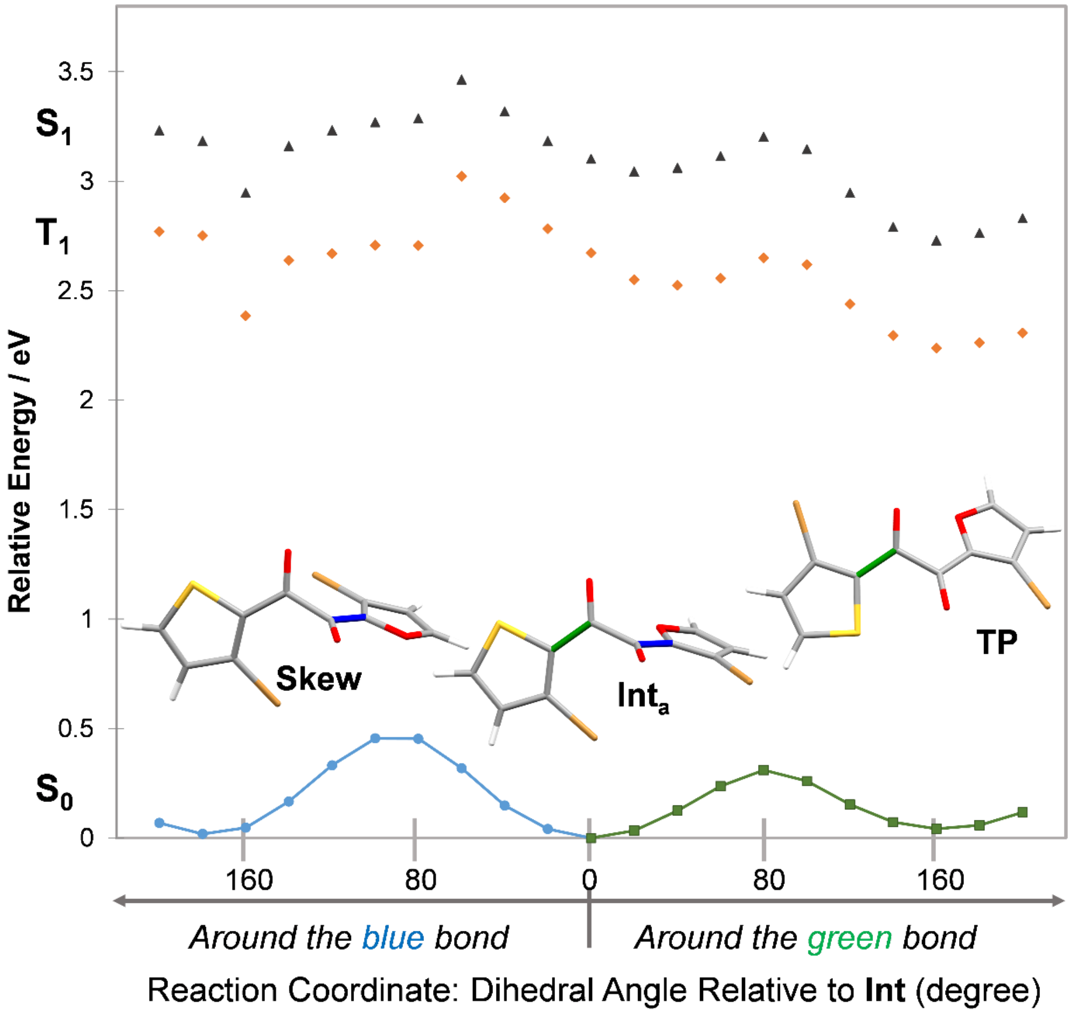

Figure S13. Potential energy curve of isolated (*S*)-**3'** along the dihedral angles of the indicated bonds calculated at the B3LYP-D3/6-311G(d) level with a step of 20°. Energies for S_1 (black triangle) and T_1 states (orange diamond) were calculated for vertical excitation.

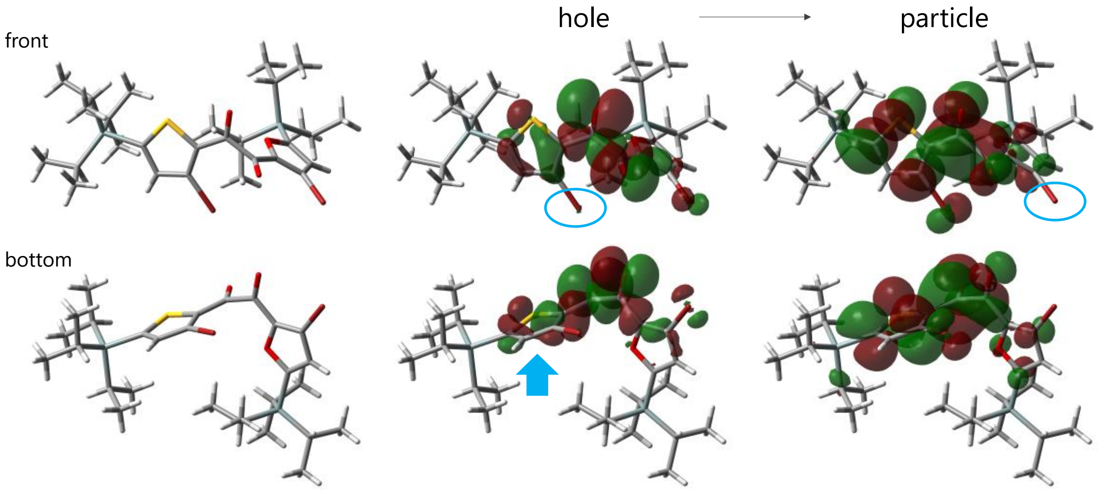

Figure S14. Molecular structure and natural transition orbitals for the phosphorescence (T_1–S_0) transition at the geometry of the crystal structure, calculated at the TD-uB3LYP-D3/6-311G(d) level of theory. Blue ellipses highlight the absence of orbital on bromine atoms, indicative of an inefficient heavy atom effect. The blue arrow highlights a π-symmetrical orbital contribution on the thiophene ring, which contaminates into the ^{3}(n,π*) transition and decreases the transition probability.

6. Photophysical and structural properties of solid 3

PLQY of solid 3

We were unable to determine the PLQY of solid **3** because no emission was detected at all using the integrating sphere equipped on the C11347-01 spectrometer (Figure S15).

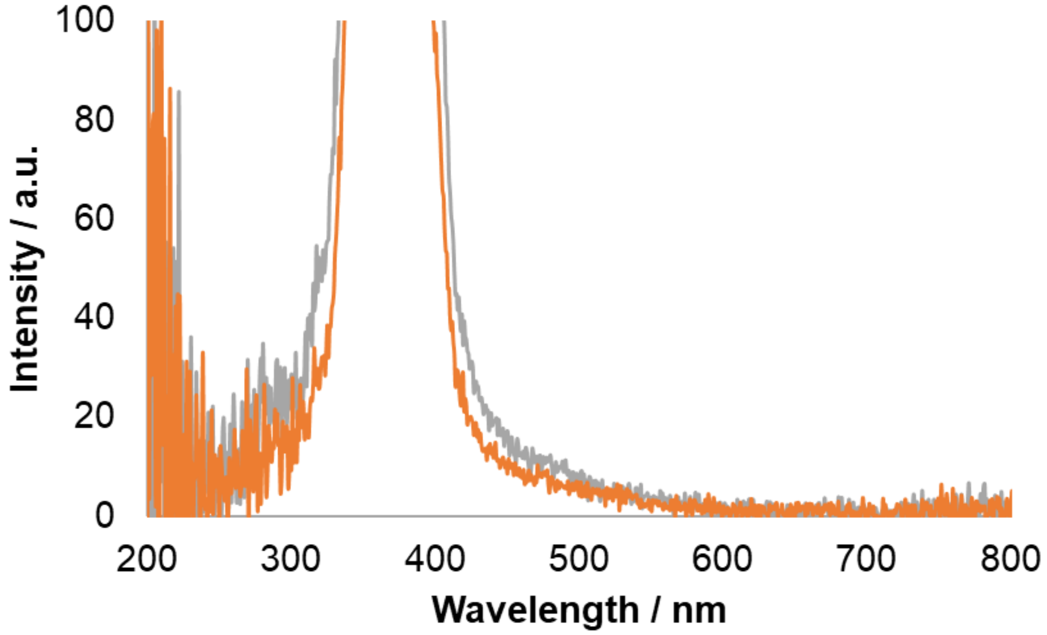

Figure S15. PL spectrum of solid **3** measured using an integrating sphere (gray: blank, orange: solid sample, λ_{ex} = 368 nm).

Single Crystal X-ray Structure Analysis

Data were collected on a Rigaku XtaLAB Synergy-R diffractometer with graphite monochromated MoKα radiation (λ = 0.71075 Å) in the ω-scan mode. The crystal was cooled by a stream of cold N_2 gas. Collection, indexing, peak integration, cell refinement, and scaling of the diffraction data were performed using the CrysAlisPro (Rigaku) software. The structure was solved by direct method (SHELXT 2018) and refined by full-matrix least-square refinement on F^2 (SHELXL2018). The non-hydrogen atoms were refined anisotropically. All hydrogen atoms were placed on the calculated positions and refined using the riding model.

Table S2. Crystallographic Data for **3** (CCDC 2095928)

Empirical formula	$C_{28}H_{44}Br_2O_3SSi_2$
Formula weight	676.69
Temperature / K	123
Crystal system	Monoclinic
Space group	$P\ 2_1/n$
a / Å	14.1498(4)
b / Å	9.2174(3)
c / Å	25.7482(8)
α / °	90
β / °	95.165(3)
γ / °	90
Volume / $Å^3$	3344.56(18)
Z	4
ρ(calcd) / $g·cm^{-3}$	1.344
Crystal size / mm^3	0.4 × 0.2 × 0.1
Completeness	99.99%
Goodness-of-fit on F^2	1.02
Final R indexes [I > =2σ (I)]	R_1 = 0.0569, wR_2 = 0.1030
Final R indexes [all data]	R_1 =0.0958, wR_2 = 0.1135
CCDC number	2095928

Hirshfeld surface analysis of 1 and 3 crystals

Hirshfeld surface analysis provides a way to view molecules as "organic wholes".[10a] To investigate the intermolecular interactions visually and quantitatively, Hirshfeld surfaces of **1** and **3** in the crystal structure were mapped with normalised contact distance d_{norm} with a fixed color scale of –0.3 (red) to 1.7 (blue) using CrystalExplorer21 software.[10b] d_{norm} is defined as follows:

$$d_{norm} = \frac{d_i - r_{vdW}}{r_{vdW}} + \frac{d_e - r_{vdW}}{r_{vdW}}$$

where d_{i} is the distance from the Hirshfeld surface to the nearest nucleus inside the surface, d_{e} is the corresponding distance to the nearest nucleus outside the surface, and r_{vdW} is the van der Waals radius of the atoms. The positive (blue) d_{norm} indicates a distance larger than van der Waals contact. As a whole, the surface of **3** is bluer than that of **1**, and the mean of d_{norm} is larger for **3** than **1**. In addition, the surface area is larger for **1** than **3** (609 and 570 Å^2). These results suggest weaker intermolecular interactions in **3** than **1**.

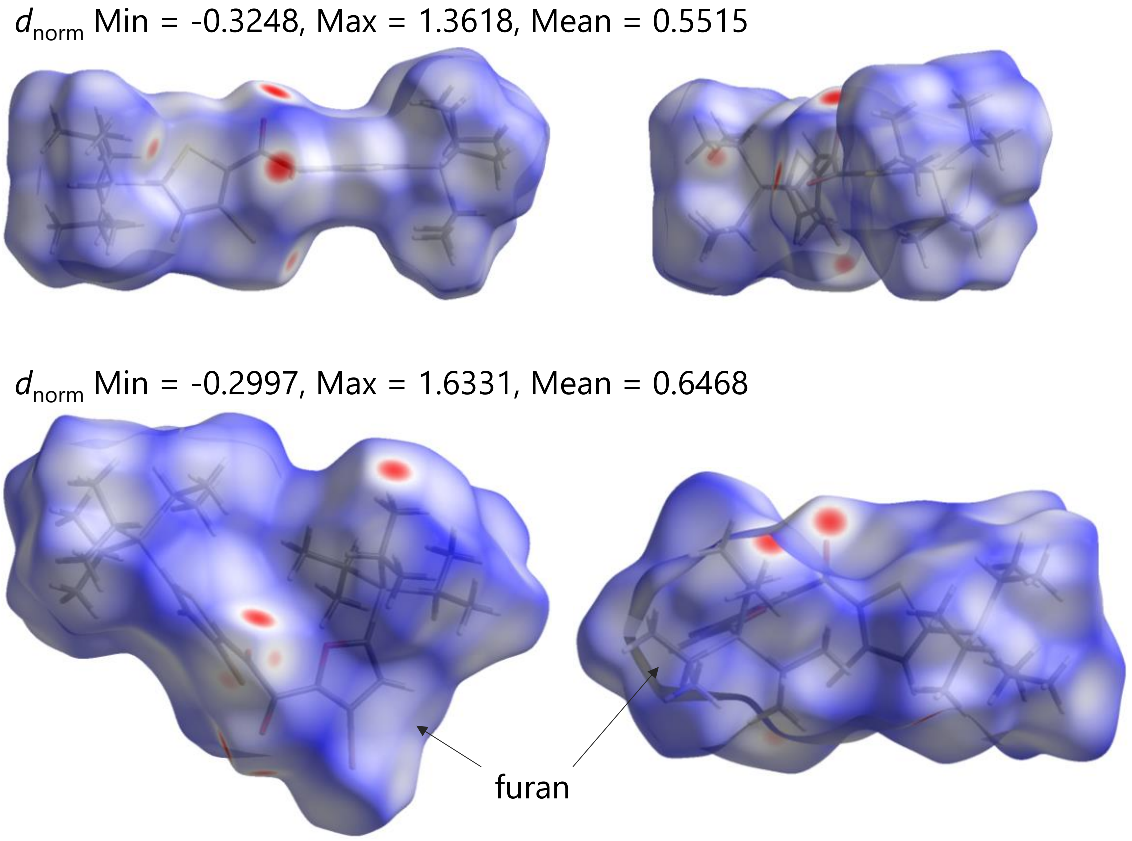

Figure S16. Hirshfeld surface of **1** (top) and **3** (bottom) mapped with d_{norm} (mapped over a fixed color scale of –0.3 (red) to 1.7 (blue)). The surfaces as well as the minima, maxima, and mean values of d_{norm} were obtained by CrystalExplorer21.

Structural identity of the single crystal and solid

PXRD patterns of solid **3** were collected and compared with those simulated from the crystal structure. Considering that the crystal structure was determined at 123 K while the PXRD of the solid was conducted at RT, these two profiles matched very well, which indicates that the molecular arrangement and conformation in the solid sample were similar to those in the crystal.

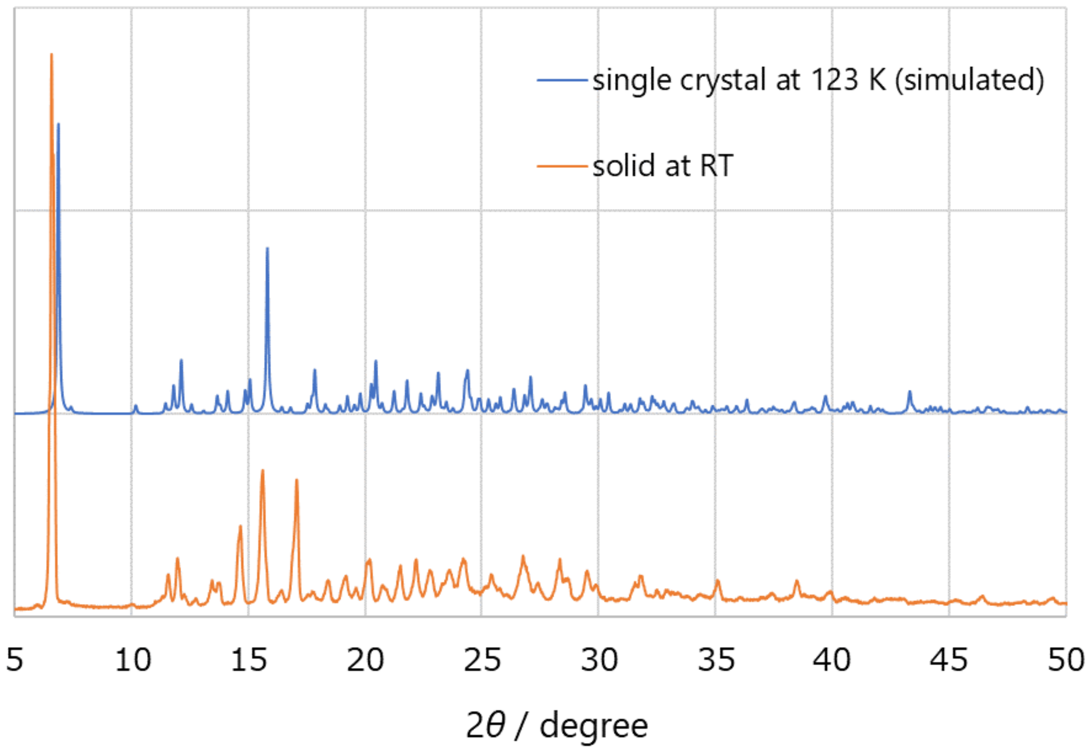

Figure S17. PXRD profiles of the single crystal of **3** (simulated, blue line) and solid **3** (orange line).

7. Thermal property and thermochromic behavior of 3

Differential scanning calorimetry (DSC)

DSC was performed with a Hitachi NEXTA DSC200 (Figure 6c and Figure S18, the same measurement) or a Rigaku Thermo Plus EVO II (Figure S19) under a flow of nitrogen. There is no exothermic recrystallization peak in the heating and cooling traces even at slower scanning rates (up to 0.3 °C·min^{-1}).[11]

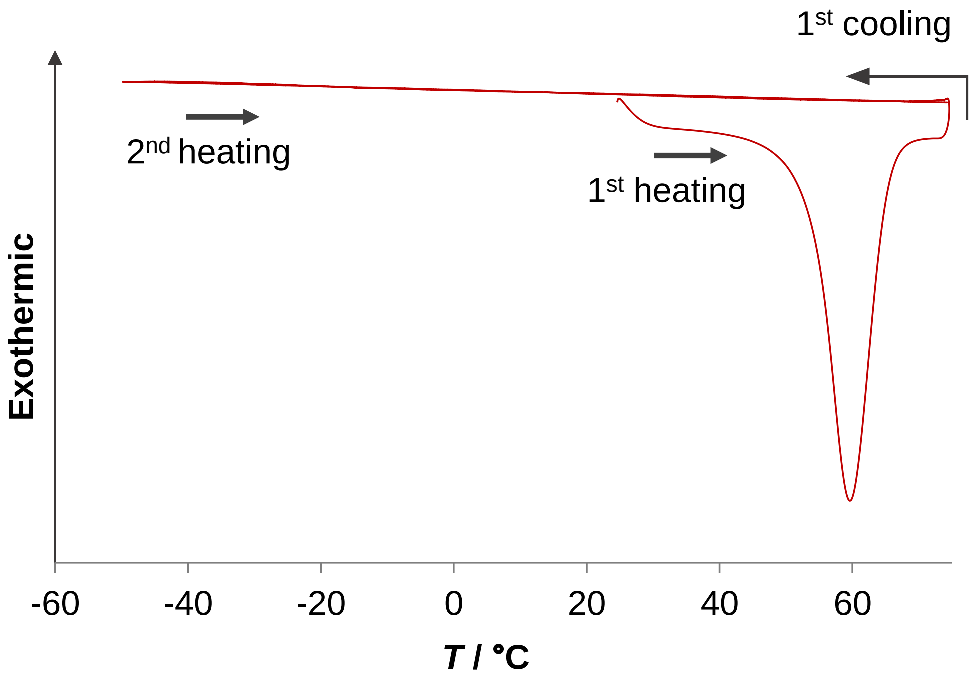

Figure S18. DSC thermograms of solid **3** during heating/cooling cycles at 10 °C·min^{-1} for the 1st heating and at 0.3 °C·min^{-1} for the 1st cooling and 2nd heating processes, under a flow of N_2.

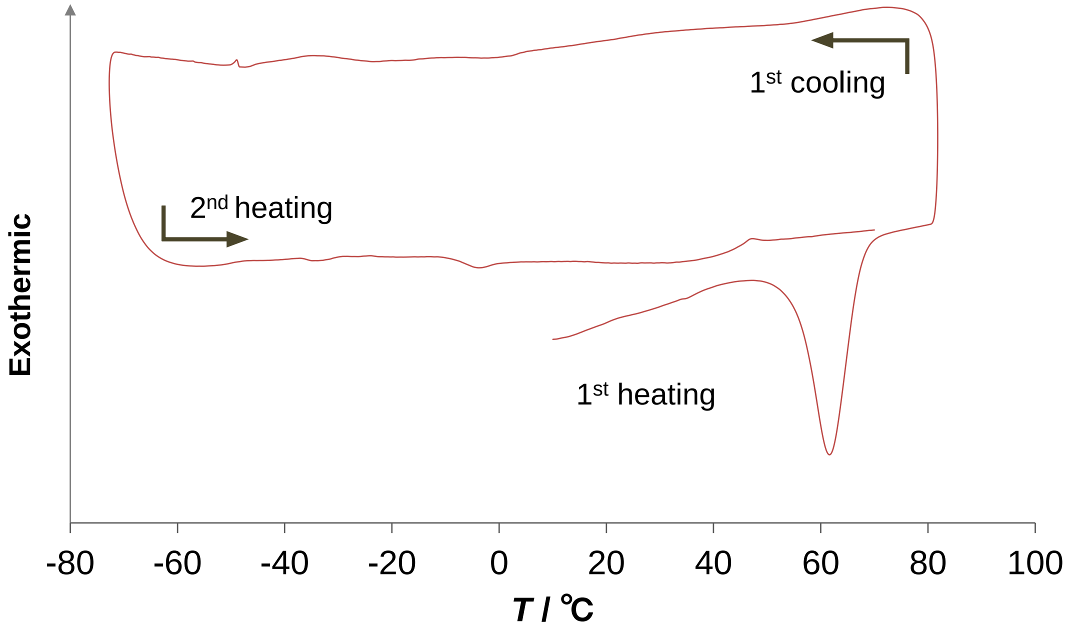

Figure S19. DSC thermograms of solid **3** during heating/cooling cycles at 10 °C·min^{-1} under a flow of N_2.

Variable-Temperature PL measurement of the liquid 3

Liquid **3** was placed in a quartz tube and capped with a silicon septum under air. The PL spectra were acquired from –120 to 20 °C in 10 °C steps using a JASCO FP-8200 spectrometer equipped with a CoolSpeK USP-203-B cryostat (UNISOKU) with a L42 sharp cut filter (HOYA, longpass, > 420 nm) and a U340 bandpass filter (HOYA) (λ_{ex} = 368 nm). The intensity increased at a lower temperature without the emergence of new peaks, indicating that the emission is phosphorescence-dominated (Figure S20). The plot of the maximum emission wavelength (λ_{max}) against reciprocal temperature (1/T) was constructed from three independent measurements with a standard deviation as error bars (Figure 5b).

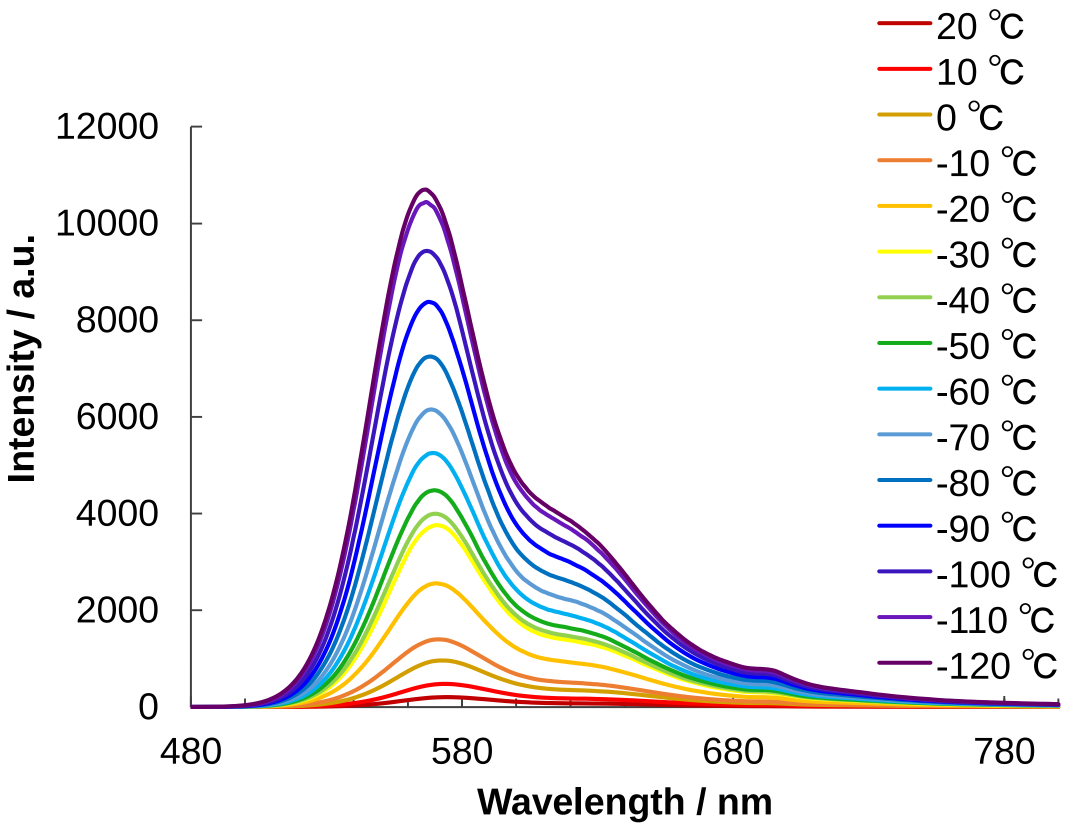

Figure S20. Steady-state photoluminescence spectra of liquid **3** acquired from –120 to 20 °C in 10 °C steps.

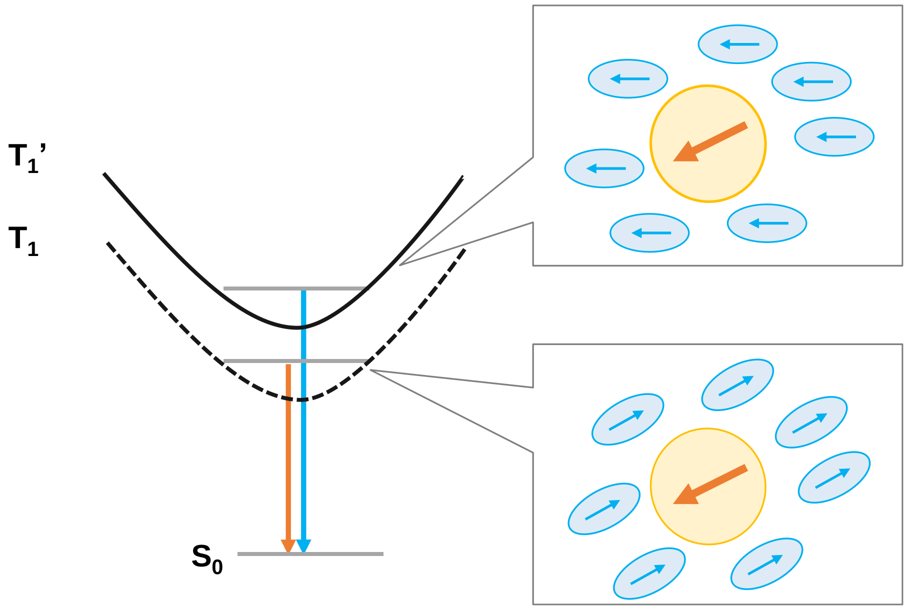

Figure S21. Schematic representations explaining the thermochromic phosphorescence: blue shift at low-temperature. At RT (broken curve, T_1), the surrounding molecules (blue

circles) reorganize to stabilize the dipole of the excited molecule (yellow circle). At low temperature, the reorganization was prevented owing to an increased viscosity, which results in the emission from a higher energy (solid curve, T_1').

8. NMR charts

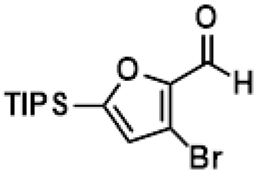

S1

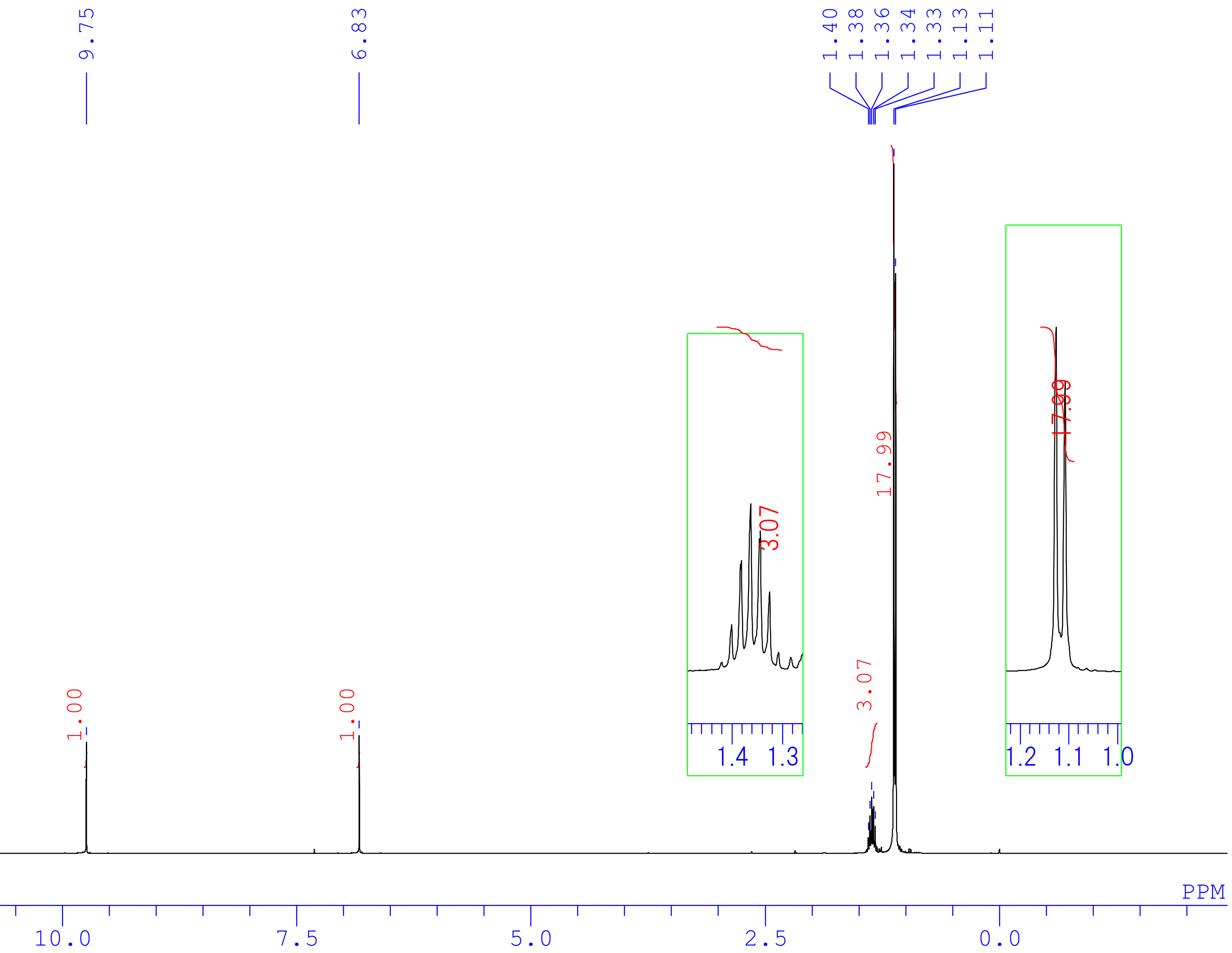

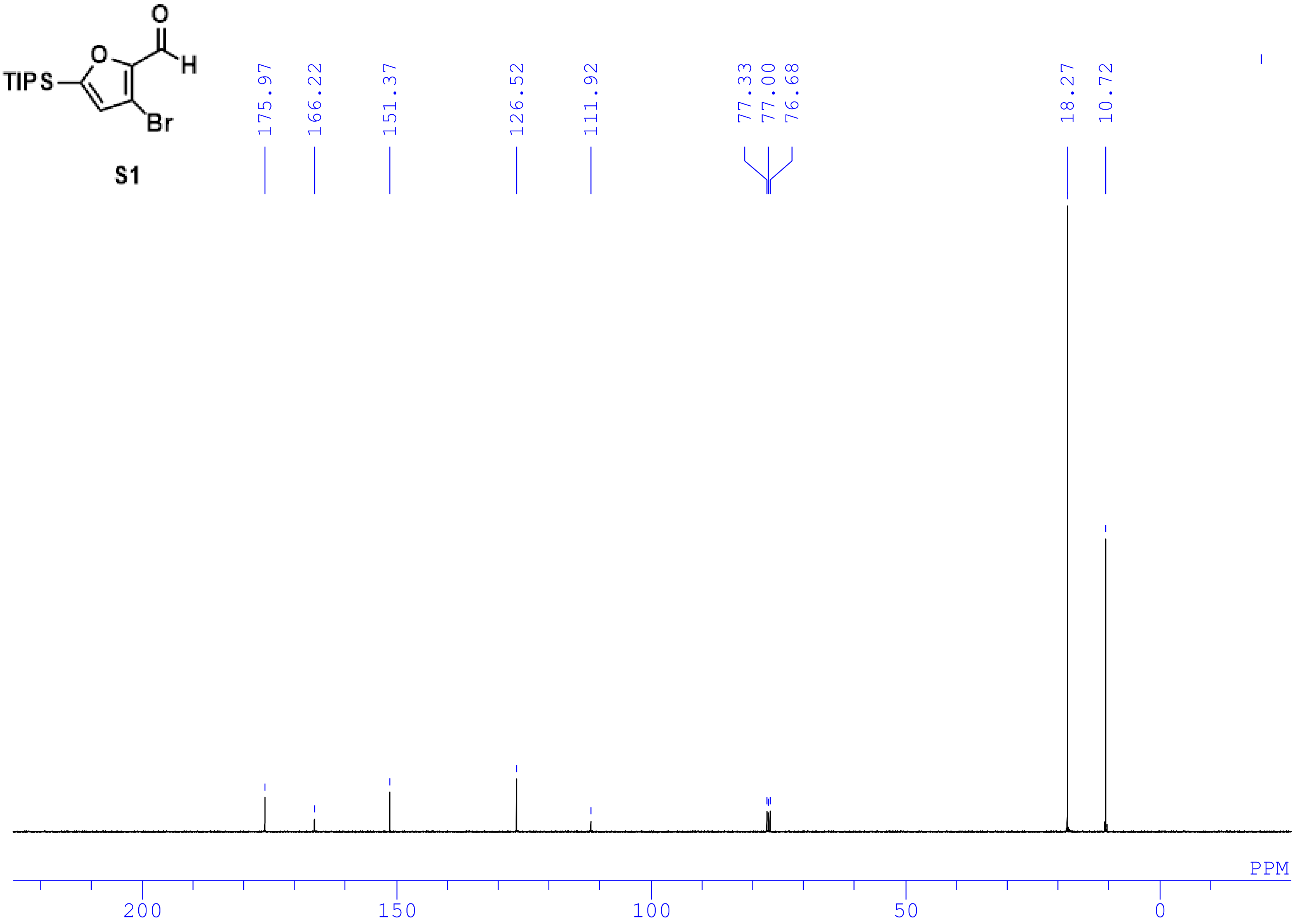
TIPS
O
H
Br
S1
175.97
166.22
151.37
126.52
111.92
77.33
77.00
76.68
18.27
10.72
PPM
200
150
100
50
0

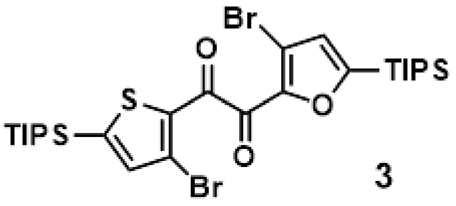
Br
O
S
TIPS
O
Br
TIPS
O

3

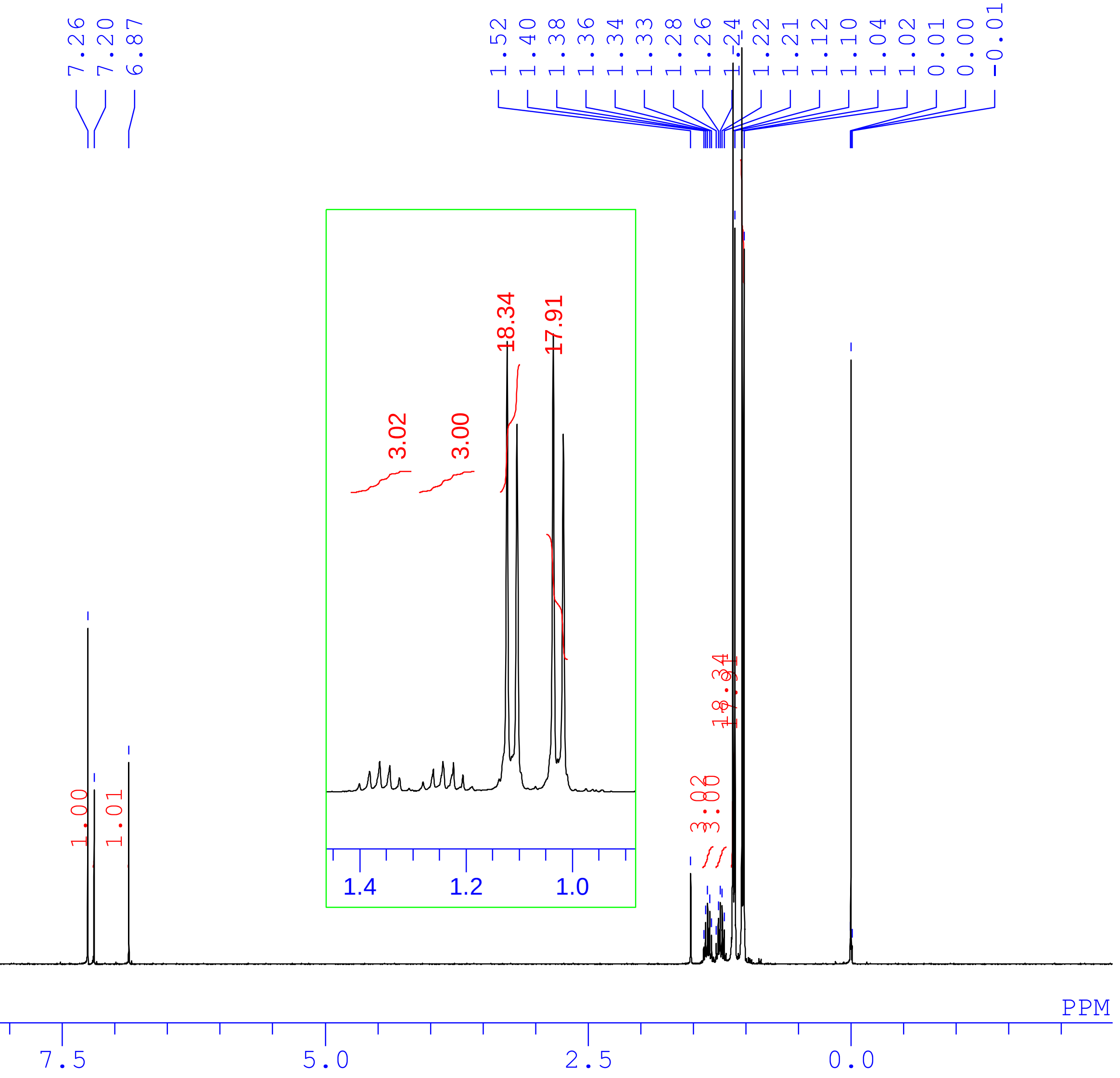
7.26
7.20
6.87
1.52
1.40
1.38
1.36
1.34
1.33
1.28
1.26
1.24
1.22
1.21
1.12
1.10
1.04
1.02
0.01
0.00
-0.01
1.00
1.01
3.02
3.00
18.34
17.91
1.4
1.2
1.0
PPM
12.5
10.0
7.5
5.0
2.5
0.0

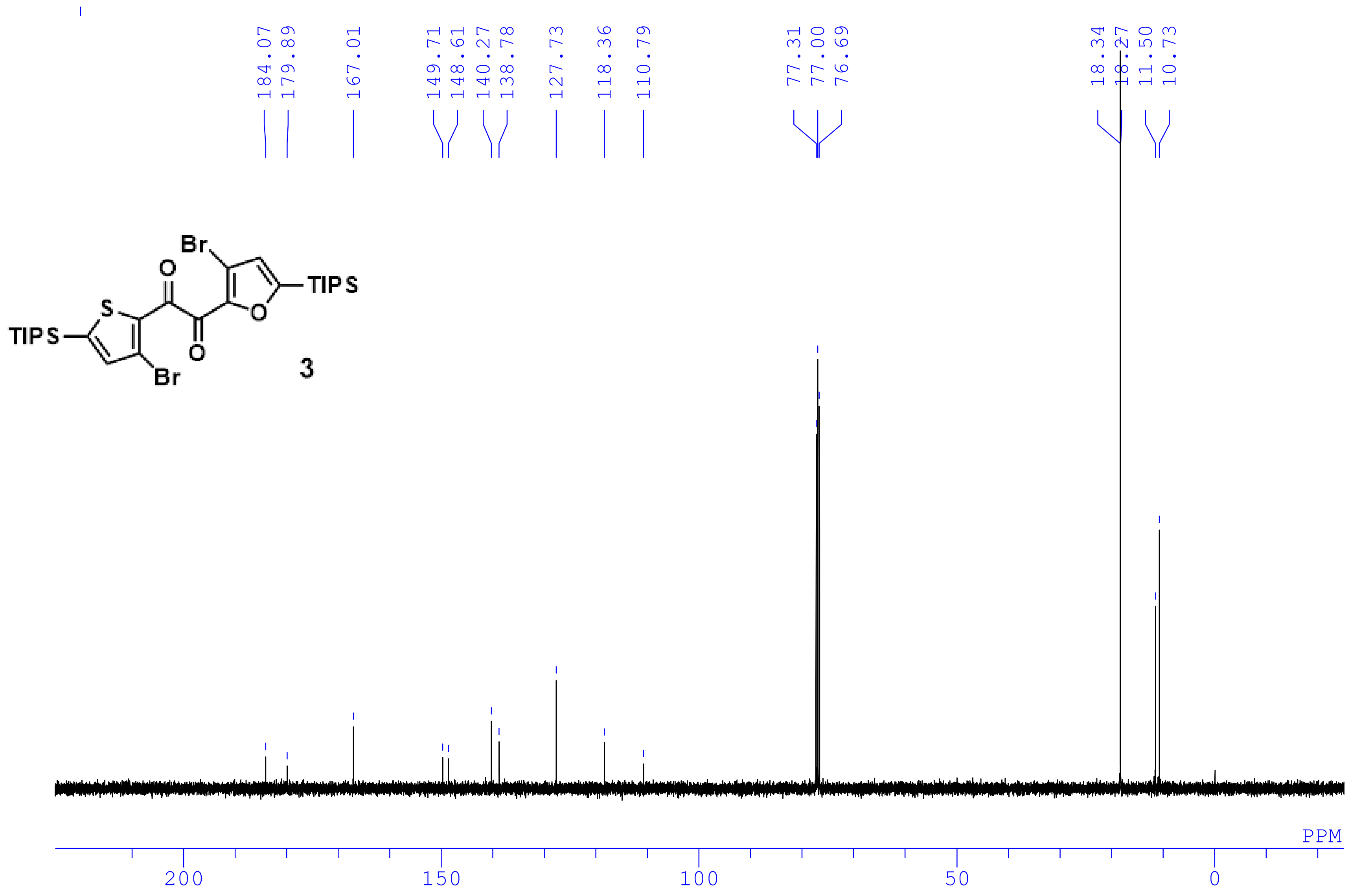
184.07
179.89
167.01
149.71
148.61
140.27
138.78
127.73
118.36
110.79
77.31
77.00
76.69
18.34
18.27
11.50
10.73
Br
O
S
TIPS
O
Br
TIPS
O
3
PPM
200
150
100
50
0

S4

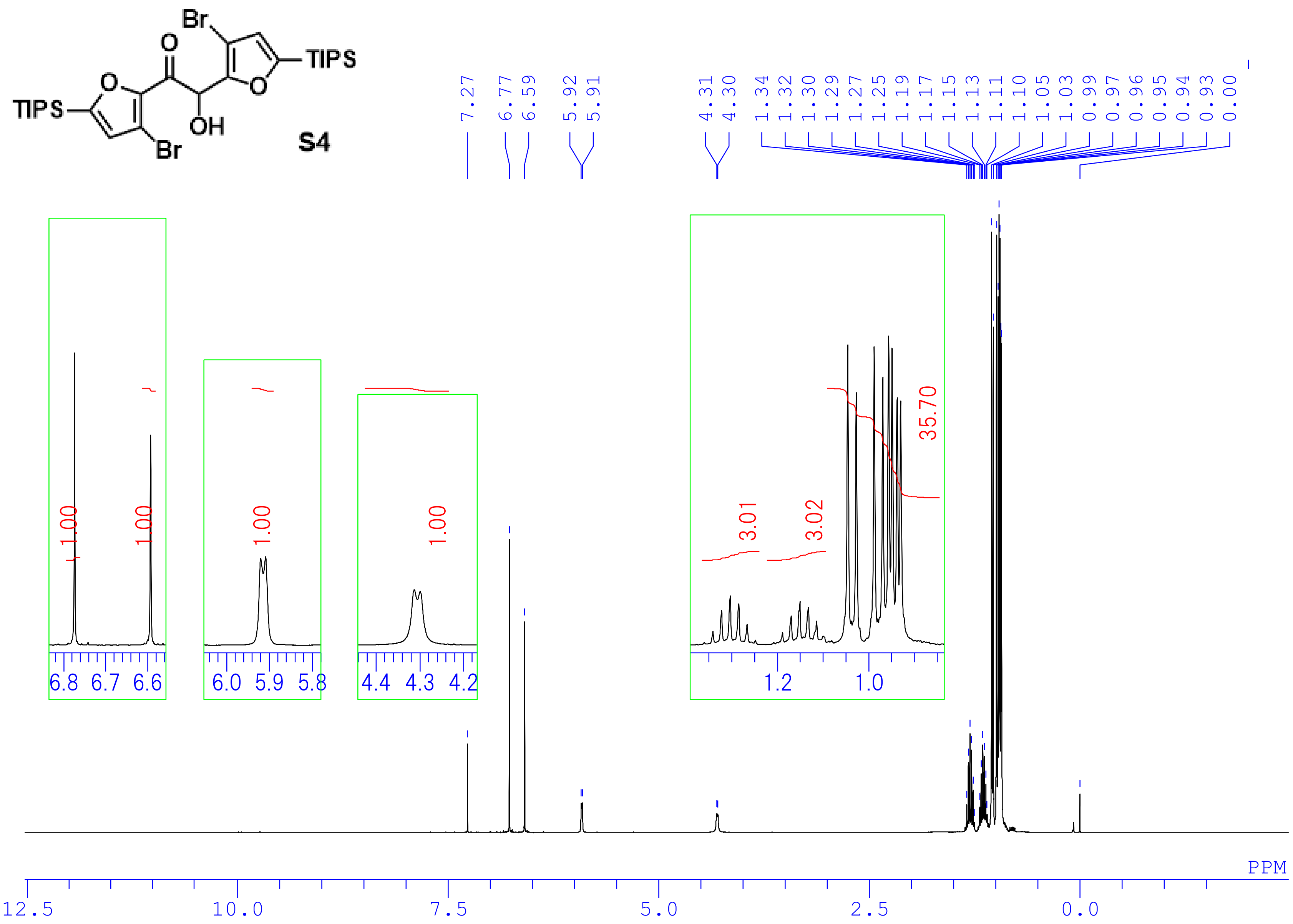

7.27
6.77
6.59
5.92
5.91
4.31
4.30
1.34
1.32
1.30
1.29
1.27
1.25
1.19
1.17
1.15
1.13
1.11
1.10
1.05
1.03
0.99
0.97
0.96
0.95
0.94
0.93
0.00
1.00
1.00
1.00
1.00
3.01
3.02
35.70
PPM
12.5
10.0
7.5
5.0
2.5
0.0

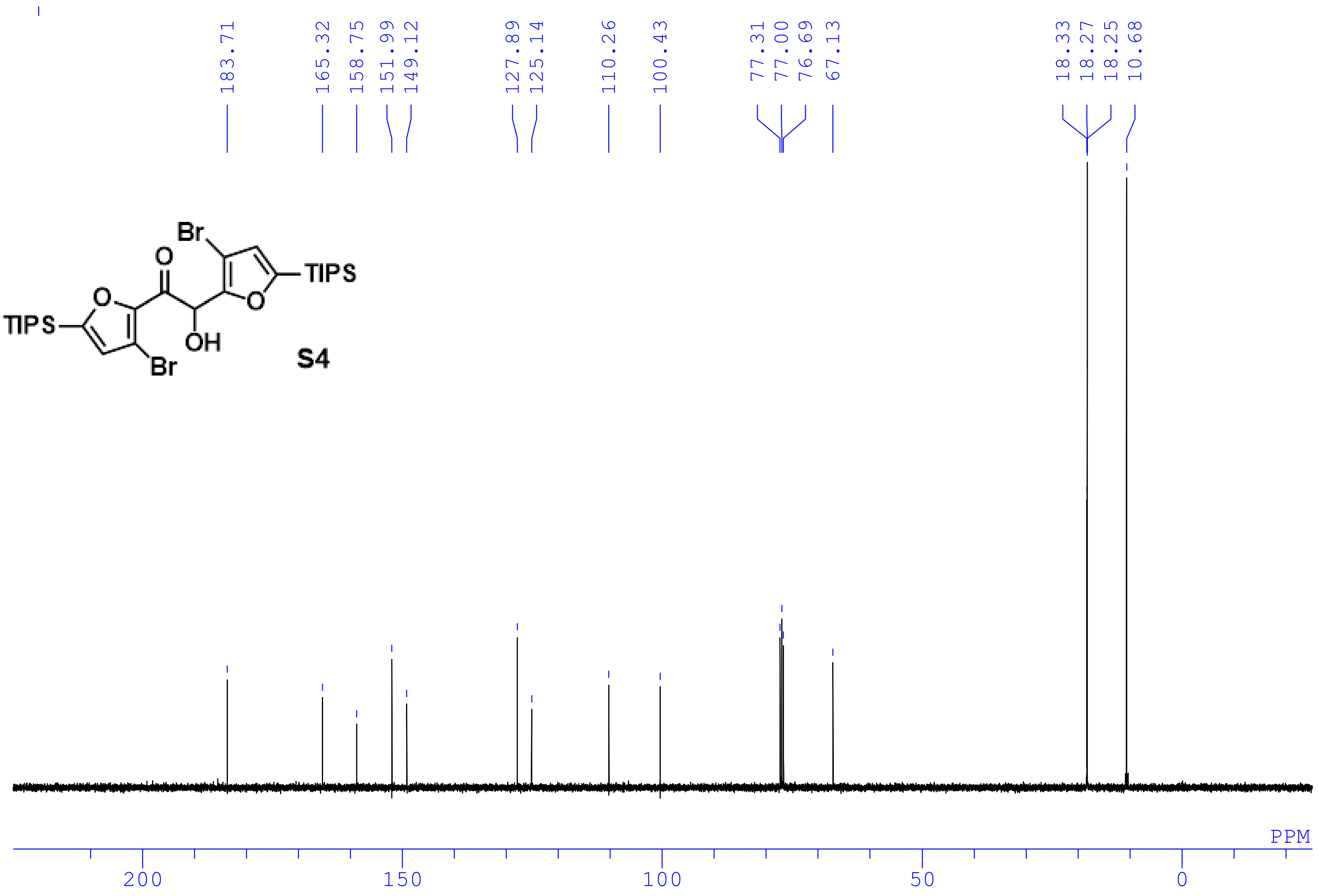
183.71
165.32
158.75
151.99
149.12
127.89
125.14
110.26
100.43
77.31
77.00
76.69
67.13
18.33
18.27
18.25
10.68
Br
O
TIPS
O
TIPS
OH
Br
S4
PPM
200
150
100
50
0

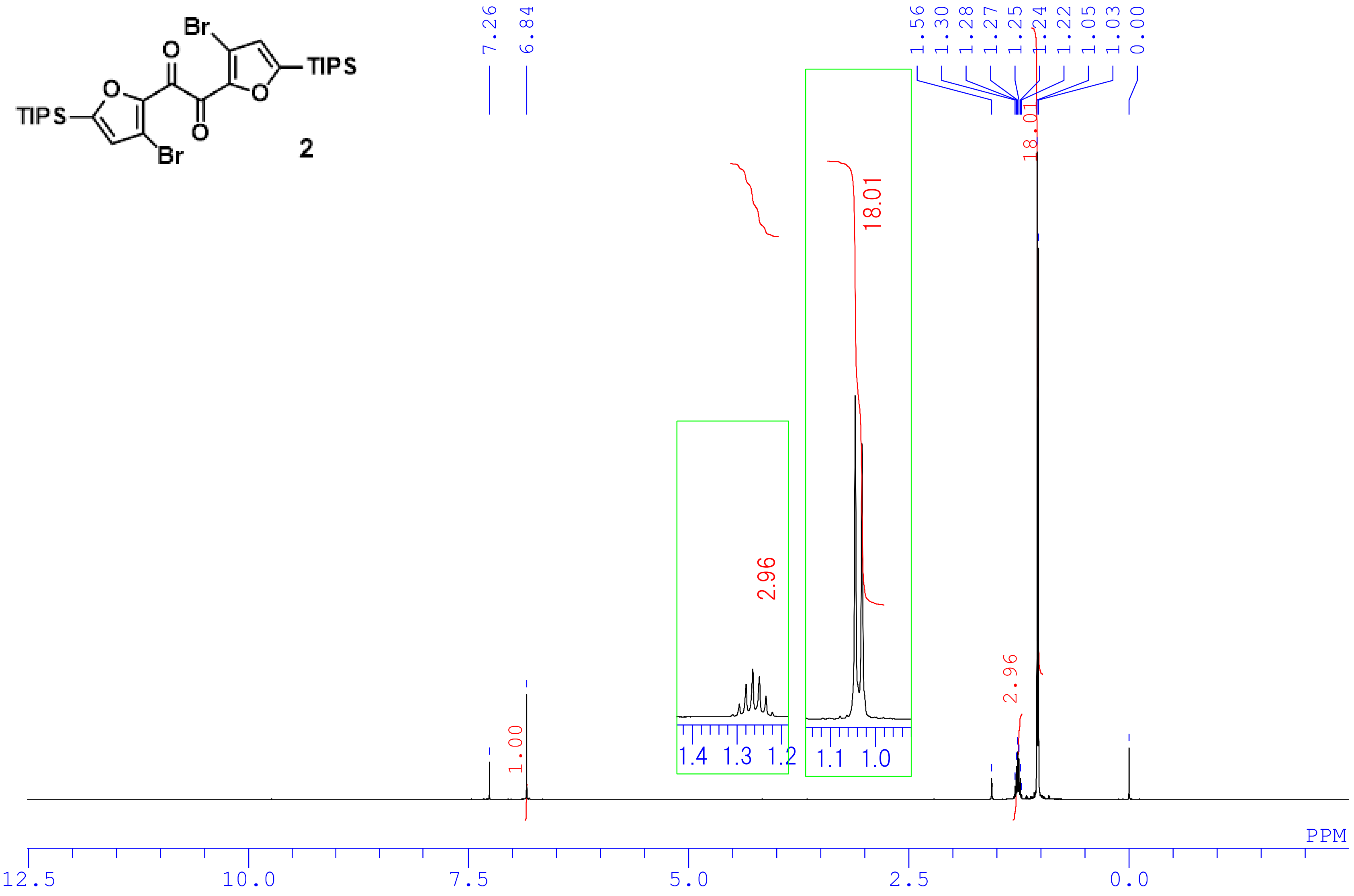
TIPS
Br
O
2
7.26
6.84
1.56
1.30
1.28
1.27
1.25
1.24
1.22
1.05
1.03
0.00
1.00
2.96
18.01
1.4 1.3 1.2
1.1 1.0
PPM
12.5
10.0
7.5
5.0
2.5
0.0

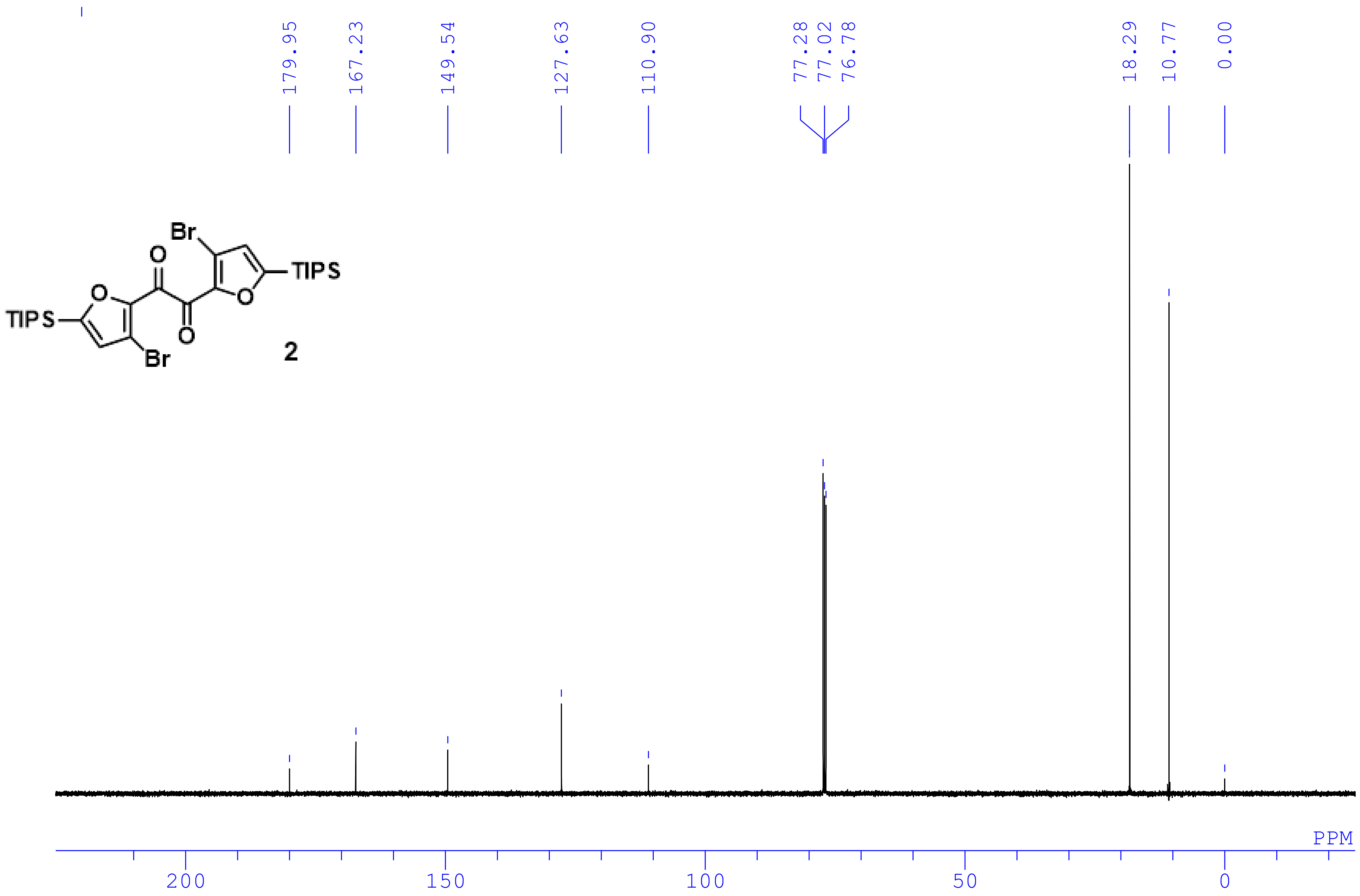

Br
O
O
TIPS
O
O
TIPS
Br
2
179.95
167.23
149.54
127.63
110.90
77.28
77.02
76.78
18.29
10.77
0.00
PPM
200
150
100
50
0

9. References

1. A. B. Pangborn, M. A. Giardello, R. H. Grubbs, R. K. Rosen, F. J. Timmers, *Organometallics* **1996**, *15*, 1518–1520.
2. (a) Y. Tani, M. Terasaki, M. Komura, T. Ogawa, *J. Mater. Chem. C* **2019**, *7*, 11926–11931. (b) Y. Tani, M. Komura, T. Ogawa, *Chem. Commun.* **2020**, *56*, 6810–6813.
3. (a) K. Suzuki, A. Kobayashi, S. Kaneko, K. Takehira, T. Yoshihara, H. Ishida, Y. Shiina, S. Oishi, S. Tobita, *Phys. Chem. Chem. Phys.* **2009**, *11*, 9850–9860. (b) A. M. Brouwer, *Pure Appl. Chem.* **2011**, *83*, 2213–2228.
4. a) D. Mari, N. Miyagawa, K. Okano, A. Mori, *J. Org. Chem.* **2018**, *83*, 14126–14137; b) J. Fröhlich, C. Hametner, *Monatsh Chem* **1996**, *127* , 435–443.
5. B. Tang, C. D. Bray, G. Pattenden, J. Rogers, *Tetrahedron* **2010**, *66*, 2492–2500.
6. E. G. Delany, S. J. Connon, *Org. Biomol. Chem.* **2018**, *16*, 780–786.
7. A. Feriani, G. Gaviraghi, G. Toson, M. Mor, A. Barbieri, E. Grana, C. Boselli, M. Guarneri, D. Simoni, S. Manfredini, *J. Med. Chem.* **1994**, *37*, 4278–4287.
8. (a) D. S. Roy, K. Bhattacharyya, S. C. Bera, M. Chowdhury, *Chem. Phys. Lett.* **1980**, *69*, 134–140. (b) K. Bhattacharyya, M. Chowdhury, *J. Photochem.* **1986**, *33*, 61–65.
9. M. J. Frisch, G. W. Trucks, H. B. Schlegel, G. E. Scuseria, M. A. Robb, J. R. Cheeseman, G. Scalmani, V. Barone, G. A. Petersson, H. Nakatsuji, X. Li, M. Caricato, A. V. Marenich, J. Bloino, B. G. Janesko, R. Gomperts, B. Mennucci, H. P. Hratchian, J. V. Ortiz, A. F. Izmaylov, J. L. Sonnenberg, Williams, F. Ding,F. Lipparini, F. Egidi, J. Goings, B. Peng, A. Petrone, T. Henderson, D. Ranasinghe, V. G. Zakrzewski, J. Gao, N. Rega, G. Zheng, W. Liang, M. Hada, M. Ehara, K. Toyota, R. Fukuda, J. Hasegawa, M. Ishida, T. Nakajima, Y. Honda, O. Kitao, H. Nakai,T. Vreven, K. Throssell, J.A. Montgomery Jr., J. E. Peralta, F. Ogliaro, M. J. Bearpark, J. J. Heyd, E.N. Brothers, K.N. Kudin, V. N. Staroverov, T. A. Keith, R. Kobayashi, J. Normand, K. Raghavachari, A. P. Rendell, J. C. Burant, S. S. Iyengar, J. Tomasi, M. Cossi, J. M. Millam, M. Klene, C. Adamo, R. Cammi, J. W. Ochterski, R. L. Martin, K. Morokuma, O. Farkas, J. B. Foresman, D.J. Fox, *Gaussian 16 Rev. B.01*; Wallingford, CT, 2016.
10. (a) M. A. Spackman, D. Jayatilaka, *CrystEngComm* **2009**, *11*, 19–32. (b) P. R. Spackman, M. J. Turner, J. J. McKinnon, S. K. Wolff, D. J. Grimwood, D. Jayatilaka, M. A. Spackman, *J. Appl. Cryst.* **2021**, *54*, 1006–1011.
11. F. Lu, K. Jang, I. Osica, K. Hagiwara, M. Yoshizawa, M. Ishii, Y. Chino, K. Ohta, K. Ludwichowska, K. J. Kurzydłowski, S. Ishihara, T. Nakanishi, *Chem. Sci.* **2018**, *9*, 6774–6778.
